# Multi-omics mapping of TIMP1-associated stromal–myeloid remodeling in autoimmune gastritis and gastric cancer

**DOI:** 10.1186/s41065-026-00736-0

**Published:** 2026-09-10

**Authors:** Jialiang Cai, Jie Li, Shu Tian, Ning Yang, Yi Wang, Xinmin Li, Yi Zhang, Shulian Bai

**Affiliations:** 1 Department of Clinical Laboratory, Medical Sciences Research Center, University-Town Hospital of Chongqing Medical University, No. 55, Daxuecheng Middle Road, Shapingba District, Chongqing, 401331 China; 2 Key Laboratory of Clinical Laboratory Diagnostics (Ministry of Education), College of Laboratory Medicine, Chongqing Medical University, Chongqing, 400016 China; 3 Chongqing Key Laboratory of Sichuan-Chongqing Co-construction for Diagnosis and Treatment of Infectious Diseases Integrated Traditional Chinese and Western Medicine, Department of Laboratory Medicine, Chongqing Hospital of Traditional Chinese Medicine, Chongqing, 400016 China; 4 Department of Laboratory Medicine, West China Hospital, Sichuan University Sichuan University West China Hospital Guang’an Hospital, Guang’an People’s Hospital, Guang’an, Sichuan 638000 China

**Keywords:** Autoimmune gastritis (AIG), Gastric cancer (GC), TIMP1, Cancer-associated fibroblasts (CAFs), Stromal–myeloid niche

## Abstract

**Background:**

Autoimmune gastritis (AIG) is a chronic immune-mediated atrophic disease that can induce cancer-related epithelial remodeling, although its association with gastric adenocarcinoma depends on histological and clinical modifiers. Gastric cancer (GC) progression is shaped by the tumor microenvironment (TME), including cancer-associated fibroblasts (CAFs), myeloid cells, and extracellular matrix (ECM) remodeling. Whether AIG-related mucosal remodeling shares stromal programs with GC-associated stromal–myeloid niches remains unclear.

**Methods:**

We integrated one AIG bulk-transcriptomic cohort and three GC cohorts to identify shared differentially expressed genes (DEGs), followed by LASSO, random forest and protein–protein interaction (PPI) analyses. TIMP1 was evaluated using tissue transcriptomic cohorts, serum RT-qPCR, survival analysis, immune infiltration profiling, scRNA-seq, inferCNV-based epithelial classification, CellChat ligand–receptor inference and spatial transcriptomics.

**Results:**

A total of 51 shared DEGs were identified between AIG and GC. TIMP1 was the only candidate supported by both machine-learning screening and PPI hub-gene analysis. TIMP1 was upregulated in GC tissues and serum and showed an exploratory tumor–normal ROC AUC of 0.941 in the TCGA_STAD cohort, while higher TIMP1 expression was associated with poorer overall survival. Functional analyses linked TIMP1-high GC to ECM organization, integrin binding, epithelial-mesenchymal transition (EMT), transforming growth factor-β signaling and immune infiltration. scRNA-seq of 160,812 GC cells localized TIMP1 mainly to CAF and myeloid compartments. In AIG, TIMP1-high fibroblasts displayed enhanced CAF-associated stromal and inflammatory transcriptional programs. CellChat and spatial transcriptomics further linked TIMP1-associated stromal and myeloid states to ECM-dominant communication and fibroblast-rich, myeloid-associated remodeling regions.

**Conclusions:**

TIMP1 may mark related stromal–myeloid remodeling states across AIG-associated mucosal remodeling and GC, providing a hypothesis-generating framework potentially relevant to inflammation-associated gastric tumorigenesis.

**Supplementary Information:**

The online version contains supplementary material available at https://doi.org/10.1186/s41065-026-00736-0.

## Introduction

Gastric cancer (GC) remains a substantial clinical burden, as many patients are diagnosed at an advanced stage and show markedly variable outcomes across clinical stages and populations. Globally, GC remains among the leading causes of cancer incidence and mortality, and recent nationwide data from China further show that the 5-year overall survival for stage IV disease remains low, highlighting the need for biomarkers that capture malignant progression and disease heterogeneity [1, 2].

Cancer treatment has evolved from surgery and conventional cytotoxic chemotherapy toward molecularly targeted therapies and immune-based approaches, substantially expanding the therapeutic landscape of many malignancies [3]. Nevertheless, therapeutic responses remain heterogeneous because tumor behavior is shaped not only by malignant-cell-intrinsic alterations but also by interactions with stromal, immune and extracellular-matrix components of the tumor microenvironment. These considerations have increased interest in biomarkers that capture both tumor-intrinsic and microenvironmental states.

Although GC arises from epithelial cells, tumor progression cannot be fully explained by malignant epithelial programs alone. The tumor microenvironment (TME), including cancer-associated fibroblasts (CAFs), myeloid cells, lymphocytes, endothelial cells and extracellular matrix (ECM), contributes to tumor growth, immune escape and therapeutic resistance [4]. Single-cell studies have revealed marked cellular and transcriptional heterogeneity in GC, whereas spatial transcriptomic studies have further shown that stromal and immune states are organized into regional niches. These findings highlight the need to understand GC progression from both epithelial and microenvironmental perspectives [5, 6].

Chronic inflammation and mucosal remodeling provide an important background for gastric carcinogenesis. The best-established model is Helicobacter pylori-associated gastric cancer, in which persistent infection may drive a stepwise progression from chronic gastritis to atrophy, intestinal metaplasia, dysplasia and carcinoma [7, 8]. Pathogenetically distinct from H. pylori-associated gastritis, AIG is primarily a host-driven, immune-mediated disease that predominantly affects the oxyntic mucosa of the gastric corpus and fundus, with relative sparing of the antrum [7, 9]. Immune-mediated parietal cell injury leads to hypochlorhydria or achlorhydria, intrinsic factor deficiency, impaired iron and vitamin B12 absorption, compensatory hypergastrinemia and progressive oxyntic atrophy [7]. This process reflects the loss of acid-secreting and intrinsic factor-producing parietal cells and results in a corpus-restricted atrophic and metaplastic mucosal phenotype. These pathological changes create a chronically injured mucosal field characterized by inflammation, glandular loss and metaplastic remodeling.

AIG is clearly associated with type 1 gastric neuroendocrine tumors, mainly through oxyntic atrophy, hypergastrinemia and enterochromaffin-like cell changes [7, 9]. Its relationship with gastric adenocarcinoma is less certain. Studies in strictly H. pylori-naive AIG cohorts suggest that adenocarcinoma risk may be lower than previously estimated, raising the possibility that unrecognized current or past H. pylori exposure may have contributed to earlier risk estimates [10]. In contrast, multicenter and pathological studies still report gastric adenocarcinoma in AIG patients, especially in the presence of intestinal metaplasia, elevated gastrin levels, older age or other clinical modifiers [11, 12]. Thus, AIG should not be described as an inevitable precancerous condition. A more accurate interpretation is that AIG represents a heterogeneous chronic inflammatory and atrophic mucosal background, in which adenocarcinoma risk is modified by histological and clinical factors [10–12].

Recent single-cell evidence further suggests that AIG and H. pylori gastritis may converge on cancer-related epithelial metaplastic states. Both conditions can generate an ANPEP/CD13-positive incomplete intestinal metaplasia-like population with transcriptional features related to GC [13]. In the comparative study by Hoft et al., metaplasia arising from H. pylori infection and AIG displayed broadly comparable histopathological and transcriptional features, and the incomplete intestinal metaplasia-like subtype showed the strongest transcriptional relatedness to GC [13]. Other pathological and molecular studies of AIG have also described marked epithelial plasticity, intestinal transdifferentiation and aberrant DNA methylation, supporting the view that autoimmune mucosal injury can reshape the gastric epithelial field [14, 15]. However, these data mainly support convergence at the level of epithelial metaplasia. They do not indicate that AIG and H. pylori infection carry equivalent cancer risk, nor do they establish a complete causal pathway from AIG to GC. Most current AIG studies still focus on parietal cell loss, epithelial atrophy, metaplasia and enterochromaffin-like cell changes. Whether AIG-associated chronic inflammatory mucosal remodeling is also accompanied by stromal remodeling remains less defined.

The stromal compartment is relevant because CAFs and myeloid cells often cooperate in GC. CAFs are not a uniform cell type; they can adopt matrix-producing, myofibroblastic, inflammatory, antigen-presenting and immunomodulatory programs depending on the tissue context and tumor state [16–18]. CAFs can produce collagens, fibronectin, laminins and inflammatory mediators, thereby shaping ECM remodeling, tissue stiffness, invasion and immune regulation [16–18]. Dense matrix architecture can also restrict immune-cell localization and contribute to immune exclusion, although such effects are context-dependent and require tumor-specific validation [19]. Myeloid populations, including tumor-associated macrophages and myeloid-derived suppressor cell-like populations, can reinforce inflammatory and immunosuppressive states [20]. Spatial transcriptomic studies have shown that fibroblast-enriched GC niches may coexist with malignant EMT programs and macrophage activation [6, 21]. Lee et al. further showed that CCL2-positive fibroblasts and STAT3-activated macrophages are spatially co-localized, associated with unfavorable prognosis, and experimentally linked to myeloid recruitment and macrophage STAT3 activation [6]. These findings support the concept that stromal-myeloid remodeling niches may be important structures in GC progression. However, whether related stromal–myeloid remodeling features are detectable in AIG mucosa remains unclear.

Tissue inhibitor of metalloproteinases-1 (TIMP1) is classically recognized as an endogenous regulator of matrix metalloproteinases and ECM turnover, but it also has non-canonical roles in cell signaling, survival, migration and immune regulation [22]. In GC, recent work has linked the SOX9/TIMP1 axis to tumor-cell proliferation, migration, invasion, FAK/PI3K/AKT signaling and dendritic-cell maturation, suggesting that TIMP1 can participate in both malignant progression and immune modulation [23, 24]. Other studies have also connected TIMP1 with EMT-like phenotypic conversion, chemoresistance-related Wnt/β-catenin signaling and prognostic relevance in gastrointestinal malignancies [25–27]. Nevertheless, most available studies address TIMP1 from a tumor-intrinsic or bulk-tissue perspective. The cell-type-specific source, communication context and spatial organization of TIMP1 along the AIG-GC axis remain unclear.

**Fig. 1:**
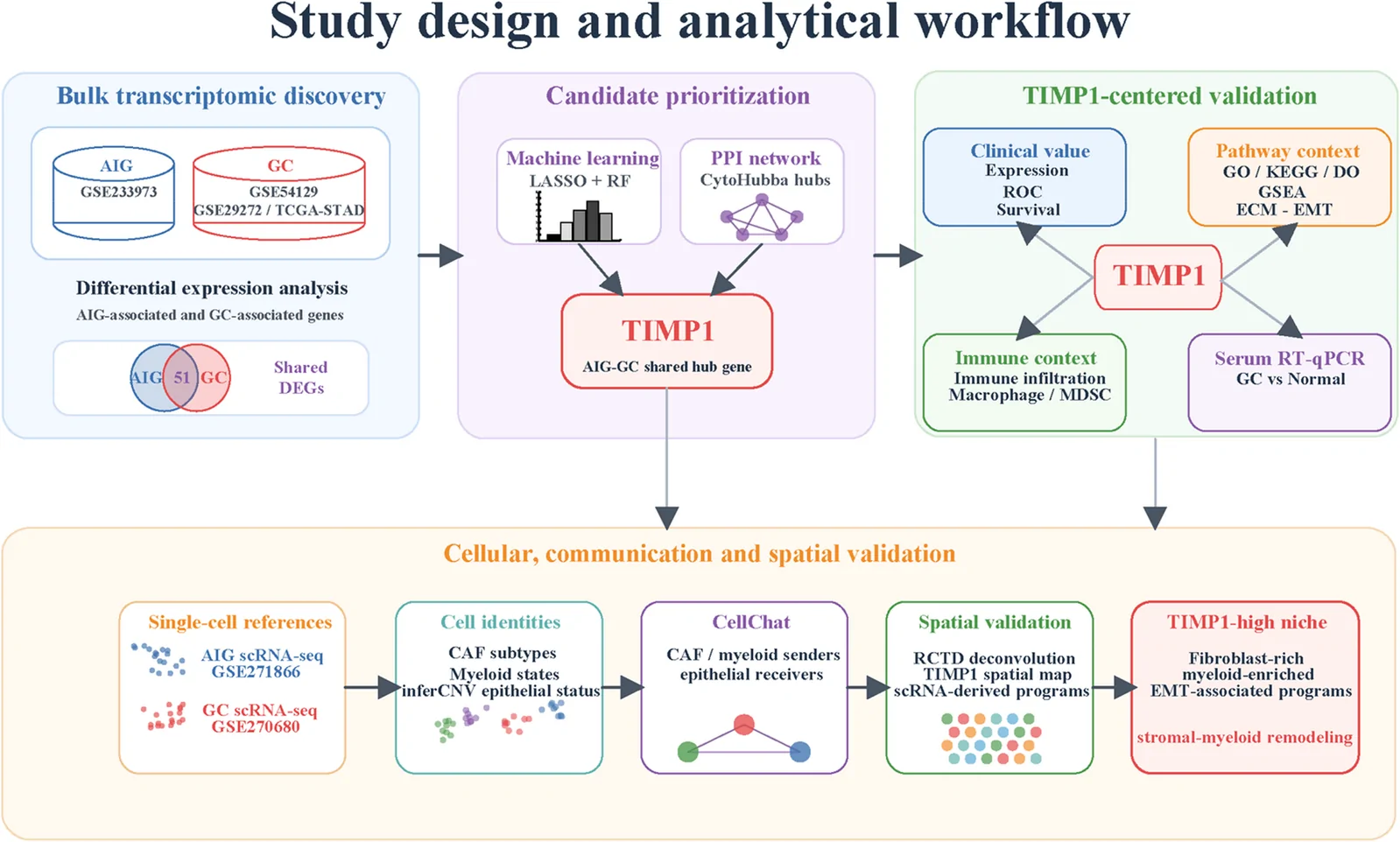
The flowchart of the study. TCGA, The Cancer Genome Atlas; GEO, Gene Expression Omnibus; GC, gastric cancer; AIG, autoimmune gastritis; STAD, stomach adenocarcinoma

We hypothesized that AIG-associated chronic inflammatory mucosal remodeling and GC-associated tumor microenvironmental remodeling may share related molecular and cellular programs. To investigate this possibility, we integrated bulk transcriptomics, machine-learning-based feature prioritization, clinical analyses, single-cell RNA sequencing and spatial transcriptomics. We first identified shared DEGs between AIG and GC and prioritized candidate molecules through cross-cohort transcriptomic and network-based analyses. TIMP1 subsequently emerged for multi-layer characterization of its expression pattern, cellular source, functional programs, epithelial communication context and spatial organization. The principal aim was not to introduce a new computational framework, but to define the cellular and spatial context of TIMP1-associated stromal–myeloid remodeling across AIG and GC and to generate a testable biological framework for inflammation-associated gastric remodeling. The overall workflow is shown in Fig. 1.

## Methods

### Data acquisition and preprocessing

Transcriptomic datasets were retrieved from the Gene Expression Omnibus (GEO, https://www.ncbi.nlm.nih.gov) repository and The Cancer Genome Atlas (TCGA, https://portal.gdc.cancer.gov/) database. Specifically, three Gastric Cancer (GC) datasets (GSE54129, *n* = 111 tumors, *n* = 21 normal; GSE29272, *n* = 134 tumors, *n* = 134 normal; and TCGA_STAD, *n* = 412 tumors, *n* = 36 normal) and one Autoimmune Gastritis (AIG) dataset (GSE233973, *n* = 14 AIG, *n* = 9 normal) were integrated for discovery [14, 28]. GEO expression matrices were processed using the limma R package. Quantile normalization was applied, with log2(x + 1) transformation performed when indicated by the expression-value distribution. TCGA_STAD raw count data were retained for DESeq2 differential-expression analysis, whereas Transcripts Per Million (TPM) values were calculated for expression-level and survival-related downstream analyses. The GSE84437 dataset was used as an independent cohort for external survival validation [29]. The detailed dataset characteristics are summarized in Table 1.

**Table 1:** Details of TCGA&GEO data used in this study

TCGA & GEO numbers	Experimental group	Control group
GSE54129	111	21
GSE29272	134	134
GSE233973	14	9
TCGA_STAD	412	36

### Identification of differentially expressed genes (DEGs)

Probes were mapped to gene symbols, and probes with ambiguous or missing gene annotations were removed. For duplicated gene symbols, one representative annotated probe was retained. Differential-expression analysis of the GEO datasets was performed using the limma package, whereas TCGA_STAD raw count data were analyzed using DESeq2. In all four discovery cohorts, significant DEGs were defined using a unified threshold of |log2 fold change| > 1 and Benjamini–Hochberg-adjusted *P* < 0.05. Overlapping DEGs across the four cohorts were subsequently identified and visualized using the ggvenn and ggsci R packages.The detailed dataset characteristics are summarized in Table 1.

### Functional and disease enrichment analysis

To elucidate the biological implications of the identified DEGs, Gene Ontology (GO), Kyoto Encyclopedia of Genes and Genomes (KEGG), and Gene Set Enrichment Analysis (GSEA) were conducted via the clusterProfiler package. GSEA utilized the c2.cp.all.v2022.1.Hs.symbols.gmt and c5.all.v2022.1.Hs.symbols.gmt reference sets. Significance was set at adj. *p* < 0.05 and False Discovery Rate (FDR) < 0.25. Disease Ontology (DO) enrichment was analyzed using the WebGestalt tool to map DEGs to specific pathological states [30].

### PPI network construction and hub gene identification

The STRING database (v12.0) was used to construct a protein–protein interaction (PPI) network with a medium confidence score of 0.400. The resulting network was visualized in Cytoscape (v3.10.3). The cytoHubba plugin was used to prioritize central nodes. The Maximal Clique Centrality (MCC) algorithm was used to identify and rank the top 10 hub genes within the network.

### Machine learning-based feature prioritization

Two complementary machine-learning approaches were used for feature prioritization among the 51 shared DEGs rather than for development of a final predictive classifier. LASSO regression was performed independently in GSE29272 and GSE54129 using the glmnet package with family = “binomial” and alpha = 1. Ten-fold cross-validation was used to determine lambda.min, and genes with non-zero coefficients were retained. Features retained by LASSO in both cohorts were considered cross-cohort LASSO candidates.

Random forest analysis was independently performed in GSE29272 and GSE54129 using the randomForest package with 1,000 trees. Gene importance was ranked according to mean decrease in Gini, and the top 10 genes from each cohort were retained. Features shared between the two random forest analyses were considered cross-cohort RF candidates. No class weighting, up-sampling, down-sampling or repeated cross-validation was applied. The intersection of the cross-cohort LASSO and RF candidate sets was used for downstream biomarker prioritization. These analyses were intended for feature screening rather than estimation of an externally validated predictive model.

Exploratory single-gene ROC analyses were performed to characterize tumor–normal discrimination. The ROC curve reported in Fig. 4C was generated from the TCGA_STAD cohort. Because TCGA_STAD also contributed to the initial shared-DEG discovery analysis, this ROC estimate was interpreted as within-study apparent discrimination rather than independent diagnostic validation.

### Survival analysis and prognostic modeling

The prognostic association of TIMP1 was assessed using Kaplan–Meier survival analysis and Cox proportional hazards regression. For Kaplan–Meier analysis, patients were stratified into TIMP1-high and TIMP1-low groups using the optimal cutoff determined by the survcutpoint function in the survminer package [31]. In Cox regression, TIMP1 expression was modeled as a continuous variable using log2(TPM + 1)-transformed expression values. Univariate Cox regression was performed to evaluate TIMP1 expression together with available clinicopathological variables. Variables showing *P* < 0.05 in univariate analysis were mapped to their corresponding original clinical variables and subsequently entered into the multivariable Cox regression model. Hazard ratios (HRs), 95% confidence intervals (CIs) and P values were reported. Overall pathological stage was not entered simultaneously with the individual T, N and M components because these variables contain substantially overlapping staging information.

### Nomogram construction and evaluation

A prognostic nomogram was constructed using the variables entered into the multivariable Cox model, including age, pathological T stage, pathological N stage, pathological M stage and TIMP1 expression, to estimate 1-, 3- and 5-year overall survival probabilities. Model calibration was evaluated using calibration curves generated with the rms package.

### Single-cell RNA-seq data processing and annotation

Single-cell RNA-seq data from GC and AIG were analyzed using Seurat package. The GC dataset GSE270680 contained 26 GC samples, and the AIG dataset GSE271866 contained 3 AIG samples [13, 32]. Raw count matrices were loaded using Read10X and converted into Seurat objects. Cells with fewer than 300 detected genes, fewer than 1,000 RNA counts, or more than 20% mitochondrial transcripts were removed. Doublets were excluded using DoubletFinder with PCs 1–20, pN = 0.25, and pK = 0.09. After filtering and doublet removal, samples were merged within each dataset, resulting in an integrated GC atlas of 160,812 cells and an integrated AIG atlas of 12,442 cells.

Data were processed using Seurat, including normalization, PCA, UMAP, and clustering (resolution = 0.5), with batch correction via Harmony. Marker genes were identified using FindAllMarkers with the MAST test, requiring min.pct > 0.25 and log2 fold change > 0.5. Cell types were assigned manually according to canonical lineage markers and cluster-specific marker expression.

### CAF and myeloid subclustering

To characterize TIMP1-associated stromal and immune states in GC, CAFs and myeloid cells were separately extracted from the integrated GC scRNA-seq atlas and analyzed using Seurat, Harmony, MAST, SCP and scCustomize. The CAF subset contained 1,696 cells, and the myeloid subset contained 1,688 cells. For each subset, cells were re-normalized using NormalizeData, variable genes were selected using FindVariableFeatures, and expression values were scaled using ScaleData. Principal component analysis was performed with RunPCA, followed by Harmony batch correction.

For both CAF and myeloid subsets, neighbor graphs were constructed using the first 15 Harmony dimensions. Clusters were identified with FindNeighbors and FindClusters at a resolution of 0.5, and UMAP was used for visualization. Cluster-enriched marker genes were identified using FindAllMarkers with the MAST test, with min.pct > 0.25 and log2 fold change > 0.5. Low-confidence or contaminating clusters were removed according to marker gene expression before final annotation.

Myeloid cells were independently reclustered after Harmony integration. Cluster-enriched marker genes were identified using FindAllMarkers with the MAST test. Final myeloid-state annotation was based on differential markers together with canonical lineage/state markers, including CD1C, FCER1A and CLEC10A for cDC2; C1QA/B/C, APOE and SPP1-associated expression for C1QC_SPP1_TAM; S100A8, S100A9, S100A12 and CXCL8 for MDSC_like; LAMP3, CCR7, FSCN1 and CCL22 for LAMP3_mregDC; CLEC9A and XCR1-associated features for cDC1; GZMB and PTGDS for pDC_like; and FCGR3A, LST1 and SERPINA1 for FCGR3A_Mono.

### Pathway and transcriptional program scoring

AUCell was used to quantify pathway and cell-state program activity at single-cell resolution. For CAFs, Hallmark gene sets related to epithelial–mesenchymal transition, TGF-β signaling, IL6–JAK–STAT3 signaling, TNFA signaling via NF-κB, inflammatory response, angiogenesis, hypoxia and coagulation were retrieved using the msigdbr package. CAF-state programs were additionally quantified at the single-cell level using AUCell. Four CAF-state gene sets were manually curated based on representative marker genes used for CAF subcluster annotation in this study, including mCAF_ECM, myCAF, iCAF and apCAF. The mCAF_ECM program included POSTN, PDGFRA, FBLN1, THBS2 and VCAN; the myCAF program included RGS5, ACTA2, MYH11, TAGLN and NDUFA4L2; the iCAF program included CFD, C7, SFRP1, SFRP2 and CXCL14; and the apCAF program included CD74, HLA-DRB1, CDH19, NRXN1 and PLAT. For each program, genes absent from the normalized RNA expression matrix were removed, and only gene sets containing at least three detected genes were retained for downstream analysis. Cell-wise gene rankings were generated from the normalized RNA expression matrix using AUCell_buildRankings, and AUC scores were calculated using AUCell_calcAUC with the top 5% of ranked genes as the maximum rank threshold.

For myeloid cells, AUCell scoring was performed using Hallmark pathways related to TGF-β signaling, IL6–JAK–STAT3 signaling, TNFA signaling via NF-κB, inflammatory response, hypoxia, angiogenesis, complement and interferon-gamma response. Custom myeloid programs were defined for FCGR3A_Mono, MDSC_like, C1QC_SPP1_TAM, cDC2, cDC1, LAMP3_mregDC and pDC_like states.

To compare TIMP1-associated transcriptional programs, cells within each CAF or myeloid state were stratified into TIMP1-high and TIMP1-low groups according to the median TIMP1 expression within that state. Program score differences between TIMP1-high and TIMP1-low cells were assessed using the Wilcoxon rank-sum test, followed by the Benjamini–Hochberg correction.

### AIG fibroblast program analysis

To examine TIMP1-associated fibroblast activation in AIG mucosa, fibroblasts were extracted from the integrated AIG scRNA-seq object and divided into TIMP1-high and TIMP1-low groups according to the median TIMP1 expression level. Two manually curated CAF-associated stromal programs were evaluated: an mCAF_ECM-like program comprising POSTN, PDGFRA, VCAN, CXCL14, MFAP4 and COL1A1, and an activated fibroblast-associated program comprising CXCL12, PDGFRB, LUM, COL1A2, ACTA2 and VCAN. TGF-β signaling, IL6–JAK–STAT3 signaling and inflammatory-response programs were additionally evaluated. Genes absent from the AIG fibroblast expression matrix were removed, and only gene sets containing at least three detected genes were retained.

Cell-level program activity was quantified using AUCell. Cell-wise gene rankings were generated using AUCell_buildRankings, and AUC scores were calculated using the top 5% of ranked genes as the maximum rank threshold. Program-score distributions between TIMP1-high and TIMP1-low AIG fibroblasts were compared using the Wilcoxon rank-sum test, followed by Benjamini–Hochberg correction for multiple comparisons.

### inferCNV analysis

Large-scale copy-number alterations were inferred from scRNA-seq data using the inferCNV R package. Epithelial identity was established before inferCNV analysis using canonical epithelial markers, including EPCAM, KRT8, KRT18, KRT19 and CDH1. Raw RNA count matrices from epithelial cells were supplied to inferCNV, with B cells serving as the non-malignant reference population. inferCNV was run using a cutoff of 0.1, with denoising enabled and HMM-based CNV calling disabled.

A relative CNV score was calculated for each epithelial cell based on deviation from the inferCNV reference baseline. Following previously reported single-cell gastric cancer strategies [33], the epithelial cluster exhibiting the lowest relative CNV burden was operationally classified as the inferCNV-defined normal epithelial population, whereas the remaining epithelial clusters were classified as inferCNV-defined malignant epithelial populations for downstream analyses. These labels represent expression-based computational classifications rather than direct DNA-level confirmation of malignancy.

### Cell–cell interaction analysis

To assess cell-cell communication within the tumor microenvironment, we applied the CellChat R package, which predicts ligand-receptor interactions from scRNA-seq profiles [34]. Inferred communication networks were generated to characterize signaling interactions among annotated cell populations. The strength and number of interactions between selected cell types were visualized using the netVisual_circle function, which displays incoming and outgoing signaling relationships in a circular layout. To examine specific signaling pathways in greater detail, the netVisual_bubble function was used to generate bubble plots highlighting representative ligand-receptor pairs and pathway-associated signaling axes.

### Spatial transcriptomics analysis

Spatial transcriptomics data from GSE251950 were analyzed for two GC sections [6], s4 and s5. Spots were retained if they had 5,000–35,000 spatial UMI counts and less than 20% mitochondrial transcripts. Genes detected in fewer than 10 spots were removed. TIMP1 expression and spatial quality-control metrics were visualized using SpatialFeaturePlot.

To estimate cellular composition in spatial spots, RCTD deconvolution was performed using the integrated GC scRNA-seq atlas as the reference. Major lineage labels were harmonized from the GC scRNA-seq annotation. Cell types represented by fewer than 30 reference cells were excluded. Common genes between the scRNA-seq reference and spatial transcriptomic data were used to construct the RCTD reference and SpatialRNA objects. RCTD was run in doublet mode, and normalized cell-type weights were added back to the corresponding Seurat spatial object.

Spatial spots were then log-normalized using NormalizeData. scRNA-seq-derived remodeling programs were scored using AddModuleScore. Program gene sets were intersected with genes detected in each spatial section before scoring. Spatial patterns were visualized with SpatialFeaturePlot.

### Immune infiltration profiling

The infiltration levels of the 28 immune cell types were quantified using the single-sample GSEA (ssGSEA) algorithm. The correlation between TIMP1 expression and specific immune subsets, including Natural Killer (NK) cells, Macrophages, Mast cells and Myeloid-Derived Suppressor Cells (MDSCs), was evaluated using Spearman’s rank correlation. Differences in immune abundance between the high and low TIMP1 groups were analyzed using the Wilcoxon rank-sum test.

### Serum RT-qPCR and HPA-based protein assessment

Serum samples were retrospectively collected from 24 GC patients and 18 healthy controls. Total RNA was extracted using the SPARKeasy Whole Blood microRNA Kit (SparkJade, AC1503), followed by reverse transcription via the FastKing cDNA Kit (TIANGEN, KR116). Quantitative PCR (qPCR) was performed using a QuantStudio™ 1 Plus System. Relative TIMP1 expression was calculated using the 2-ΔΔCt method, with GAPDH as the internal control.

- TIMP1-Forward: 5’-ttggctgtgaggaatgcaca-3’, TIMP1-Reverse: 5’-ggaagcccttttcagagcct-3’
- GAPDH-Forward: 5’-caggaggcattgctgatgat-3’, and GAPDH-Reverse: 5’-gaaggctggggctcatttt-3’,

Protein expression levels and histological distribution were validated using the Human Protein Atlas (HPA) database (https://www.proteinatlas.org/).

### Statistical analysis

All computational analyses and graphical outputs were performed using R software version 4.5.1 (https://www.R-project.org). Continuous variables were compared between two groups using either the Student’s t-test or the Wilcoxon rank-sum test, depending on data distribution. Associations between TIMP1 expression and clinicopathological characteristics were evaluated using the Wilcoxon test and logistic regression analysis. Unless otherwise stated, all statistical tests were two-sided, and *p* < 0.05 was considered statistically significant.

## Results

### Integrative transcriptomic analysis identifies TIMP1 as a shared AIG–GC hub gene

**Fig. 2:**
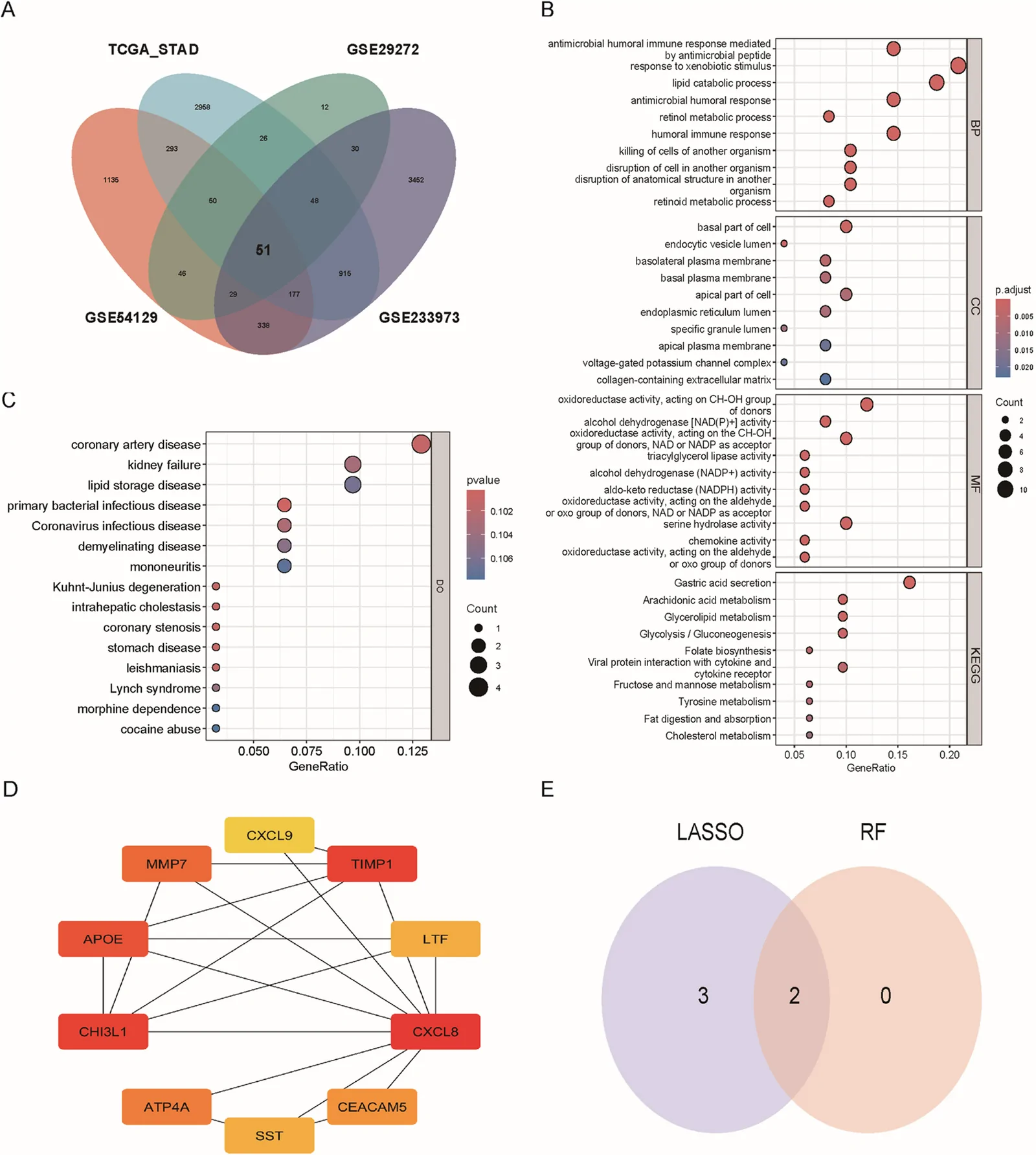
Identification and prioritization of shared transcriptomic signatures between AIG and GC. **A **Venn diagram showing the intersection of DEGs from four datasets (GSE54129, GSE29272, TCGA_STAD, and GSE233973). **B **GO and KEGG enrichment analysis of the 51 core DEGs, highlighting pathways such as gastric acid secretion and retinol metabolism. **C **DO enrichment analysis identifying associations with stomach diseases and metabolic disorders. GO, Gene Ontology; KEGG, Kyoto Encyclopedia of Genes and Genomes; DO, Disease Ontology. **D **The top 10 hub genes were examined from the PPI network of common differentially expressed genes, and based on the topological analysis of these 10 genes, the degree of CXCL8, TIMP1 and CHI3L1 was 9, 5, and 5. **E **Venn diagram showing the intersection of feature genes identified by the LASSO and RF algorithms across datasets, identifying TIMP1 and FAP

To explore shared transcriptomic alterations between autoimmune gastritis (AIG) and gastric cancer (GC), we integrated one AIG dataset and three GC datasets. Differential expression analysis identified 2,119 DEGs in GSE54129, including 1,059 upregulated and 1,060 downregulated genes; 292 DEGs in GSE29272, including 127 upregulated and 165 downregulated genes; 5,040 DEGs in GSE233973, including 2,637 upregulated and 2,403 downregulated genes; and 4,518 DEGs in TCGA_STAD, including 2,231 upregulated and 2,287 downregulated genes (Table 2). By intersecting DEGs across the four cohorts, we identified 51 shared DEGs between AIG and GC (Fig. 2A).

**Table 2:** The number of up-regulated and down-regulated genes and the number of differentially expressed genes in the differential gene analysis of each expression profile

TCGA & GEO numbers	Upregulated genes	Downregulated genes	Differential genes
GSE54129	1059	1060	2119
GSE29272	127	165	292
GSE233973	2637	2403	5040
TCGA_STAD	2231	2287	4518

We next performed functional enrichment analysis to characterize the biological relevance of these shared genes. Gene Ontology analysis showed that the 51 shared DEGs were enriched in biological processes such as the antimicrobial humoral immune response and retinol metabolic process. Cellular component terms included apical plasma membrane, basolateral plasma membrane and collagen-containing extracellular matrix, while molecular function terms were related to oxidoreductase activity and chemokine activity (Fig. 2B). KEGG pathway analysis further indicated enrichment in gastric acid secretion and arachidonic acid metabolism **(**Fig. 2B). Disease ontology analysis showed significant associations with stomach diseases, supporting the relevance of the shared DEGs to gastric pathology (Fig. 2C). Additional associations with coronary artery disease, Lynch syndrome, kidney failure, lipid storage diseases and mononeuropathy were also observed, suggesting that these genes may reflect broader inflammatory, metabolic or tissue-remodeling processes beyond gastric disease alone.

To prioritize candidate biomarkers from the 51 shared DEGs, we applied two complementary machine learning algorithms, LASSO regression and Random Forest, in the two GEO GC datasets. In the LASSO analysis, 10-fold cross-validation was used to determine the optimal penalty parameter λ. At the lambda.min value, 15 feature genes were selected in GSE29272 and 9 feature genes were selected in GSE54129 (Figure S1A–D). Random forest models were constructed using 1,000 trees, and the top 10 genes ranked by Mean Decrease Gini were retained from each dataset (Figure S1E, F). The cross-cohort LASSO and random forest analyses yielded two cross-method prioritized candidates, FAP and TIMP1 (Fig. 2E).

To further refine candidate prioritization, we constructed a protein–protein interaction network using the STRING database with a confidence score of 0.400. The top 10 hub genes were identified using the MCC algorithm in the Cytoscape plugin CytoHubba. Among these hub genes, CXCL8 showed the highest network degree, followed by TIMP1 and CHI3L1 (Fig. 2D). TIMP1 was the only machine-learning-derived candidate that was also retained among the leading PPI hub genes and was therefore selected for subsequent multi-layer biological characterization. This prioritization reflected convergence across analytical approaches rather than superiority of a single diagnostic metric.

### Multi-cohort validation of TIMP1 expression and its clinicopathological associations in GC

**Fig. 3:**
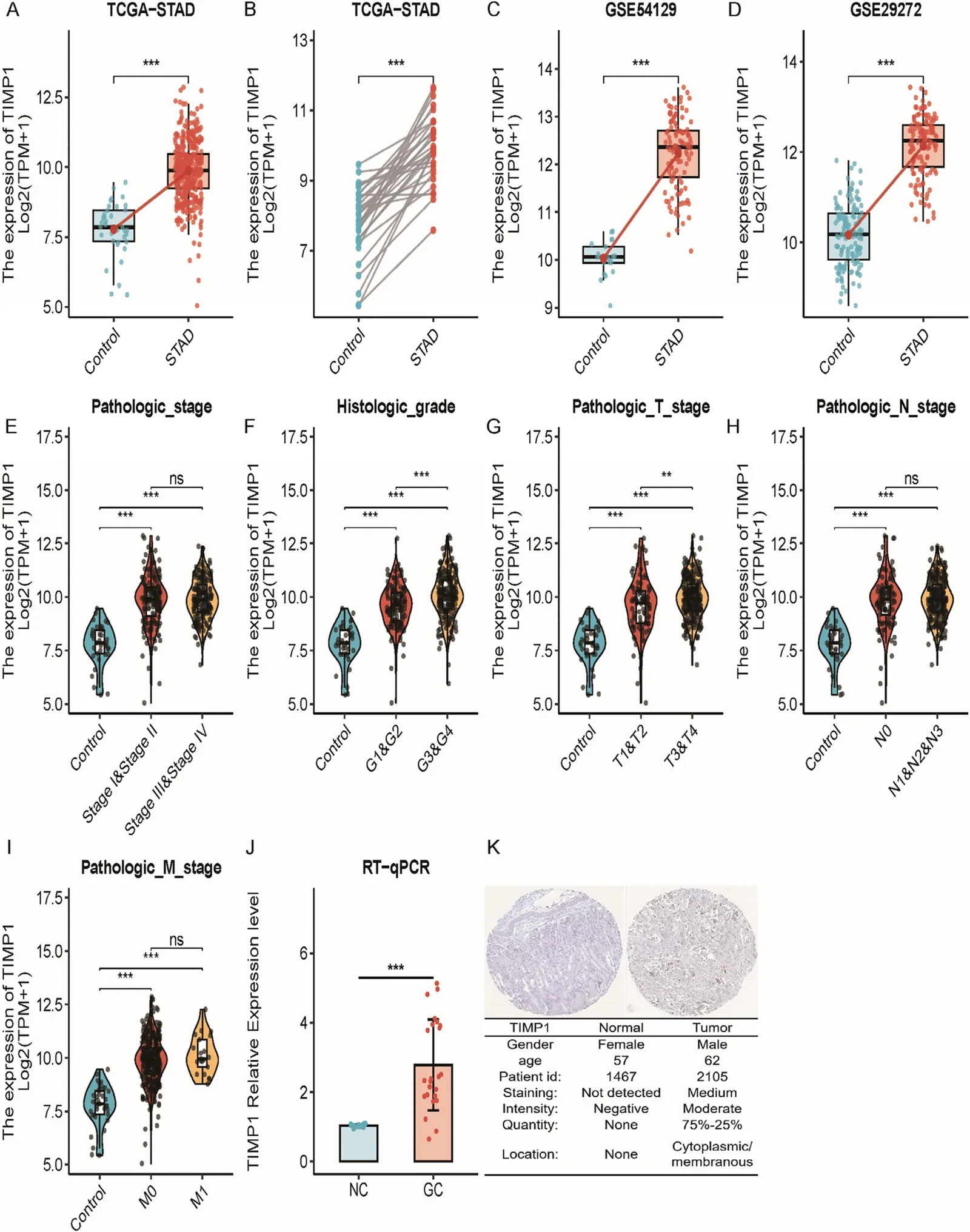
Validation of TIMP1 expression and clinical associations in GC. **A**-**D **mRNA expression levels of TIMP1 in the TCGA_STAD and GEO datasets (GSE54129, GSE29272). **E**-**I **Correlation between TIMP1 expression and clinical pathological features, including pathologic stage, T stage, histologic grade, M stage, and N stage. **J **Serum relative expression of TIMP1 in GC patients validated by RT-qPCR. **K **Validation of TIMP1 by the Human Protein Atlas database. *p < 0.05; **p < 0.01; ***p < 0.001; ns, not significant. RT-qPCR, reverse transcription quantitative polymerase chain reaction; T stage, tumor stage; G grade, histologic grade; NC, Normal Control; GC, gastric cancer

The baseline clinical and pathological characteristics of patients with STAD from the TCGA cohort are summarized in Table 3. TIMP1 expression was validated on multiple platforms. In the TCGA_STAD and GEO datasets, TIMP1 was significantly overexpressed in GC tissues compared to normal controls (*P* < 0.001) (Fig. 3A–D). Clinical correlation analysis in the TCGA cohort (*n* = 365) revealed that high TIMP1 expression was significantly associated with race (*P* < 0.01), pathologic T-stage (*P* < 0.01), and pathologic stage (*P* < 0.05) (Table 4). Specifically, TIMP1 levels significantly increased with advanced T-stage (T3&T4 vs. T1&T2, *P* < 0.01) and histologic grade (G3 vs. G1&G2, *P* < 0.001) (Fig. 3F, G). At the protein level, validation using the Human Protein Atlas (HPA) showed moderate cytoplasmic and membranous staining in 25%–75% of tumor cells, whereas TIMP1 was not detected in normal gastric tissues (Fig. 3K). In addition, serum RT-qPCR analysis in the clinical cohort (24 GC patients and 18 healthy controls) showed significantly higher TIMP1 RNA levels in GC patients (*P* ≤ 0.001; Fig. 3J), providing preliminary serum RNA-level support for elevated TIMP1 expression. These findings consistently support the upregulation of TIMP1 in GC at both the tissue and serum levels.

**Table 3:** Baseline clinical and pathological characteristics of patients with stomach adenocarcinoma (STAD) from the TCGA cohort

Characteristics	Clinical Group	Overall
OS	Alive	231 (63.3%)
	Dead	134 (36.7%)
OS_time	Mean ± SD	625.1 ± 540.8
dss	Mean ± SD	0.3 ± 0.4
dss_time	Mean ± SD	625.1 ± 540.8
pfi	Mean ± SD	0.4 ± 0.5
pfi_time	Mean ± SD	564.8 ± 540.9
Age	<=60	125 (34.5%)
	> 60	237 (65.5%)
Gender	Female	126 (34.5%)
	Male	239 (65.5%)
Race	White	230 (70.8%)
	Asian	83 (25.5%)
	Black or African American	12 (3.7%)
H. pylori infection	No	151 (88.3%)
	Yes	20 (11.7%)
Pathologic_T_stage	T1	19 (5.2%)
	T2	77 (21.1%)
	T3	165 (45.2%)
	T4	104 (28.5%)
Pathologic_N_stage	N0	113 (31.7%)
	N1	96 (26.9%)
	N2	73 (20.4%)
	N3	75 (21.0%)
Pathologic_M_stage	M0	329 (94.0%)
	M1	21 (6.0%)
pathologic_stage	Stage I	51 (14.4%)
	Stage II	116 (32.7%)
	Stage III	156 (43.9%)
	Stage IV	32 (9.0%)
Tumor status	Tumor free	267 (77.6%)
	With tumor	77 (22.4%)
Residual_tumor	R0	312 (92.3%)
	R1 & R2	26 (7.7%)
Histologic_grade	G1	9 (2.5%)
	G2	130 (36.5%)
	G3	217 (61.0%)
	G4	0 (0.0%)
Barrett’s esophagus	No	208 (92.9%)
	Yes	16 (7.1%)

**Table 4:** Relationship between TIMP1 expression and clinicopathological features in the TCGA database

Characteristics	Clinical Group	TIMP1 Low (*N* = 182)	TIMP1 High (*N* = 183)	*P* Value
OS	Alive	124 (68.1%)	107 (58.5%)	> 0.05
	Dead	58 (31.9%)	76 (41.5%)	
OS_time	Mean ± SD	657.5 ± 599.0	592.9 ± 475.4	> 0.05
dss	Mean ± SD	0.2 ± 0.4	0.3 ± 0.5	> 0.05
dss_time	Mean ± SD	657.5 ± 599.0	592.9 ± 475.4	> 0.05
pfi	Mean ± SD	0.3 ± 0.5	0.4 ± 0.5	> 0.05
pfi_time	Mean ± SD	607.9 ± 592.6	522.0 ± 481.9	> 0.05
Age	<=60	60 (33.5%)	65 (35.5%)	> 0.05
	> 60	119 (66.5%)	118 (64.5%)	
Gender	Female	63 (34.6%)	63 (34.4%)	> 0.05
	Male	119 (65.4%)	120 (65.6%)	
Race	White	102 (66.2%)	128 (74.9%)	** < 0.01
	Asian	41 (26.6%)	42 (24.6%)	
	Black or African American	11 (7.1%)	1 (0.6%)	
H_pylori_infection	No	102 (91.9%)	49 (81.7%)	> 0.05
	Yes	9 (8.1%)	11 (18.3%)	
Pathologic_T_stage	T1	17 (9.3%)	2 (1.1%)	** < 0.01
	T2	40 (22%)	37 (20.2%)	
	T3	71 (39%)	94 (51.4%)	
	T4	54 (29.7%)	50 (27.3%)	
Pathologic_N_stage	N0	56 (31.5%)	57 (31.8%)	> 0.05
	N1	38 (21.3%)	58 (32.4%)	
	N2	44 (24.7%)	29 (16.2%)	
	N3	40 (22.5%)	35 (19.6%)	
Pathologic_M_stage	M0	164 (94.8%)	165 (93.2%)	> 0.05
	M1	9 (5.2%)	12 (6.8%)	
pathologic_stage	Stage I	34 (19.2%)	17 (9.6%)	* < 0.05
	Stage II	46 (26%)	70 (39.3%)	
	Stage III	81 (45.8%)	75 (42.1%)	
	Stage IV	16 (9%)	16 (9%)	
Tumor_status	Tumor free	128 (73.6%)	139 (81.8%)	> 0.05
	With tumor	46 (26.4%)	31 (18.2%)	
Residual_tumor	R0	160 (92.5%)	152 (92.1%)	> 0.05
	R1 & R2	13 (7.5%)	13 (7.9%)	
Histologic_grade	G1	4 (2.3%)	5 (2.8%)	> 0.05
	G2	82 (46.3%)	48 (26.8%)	
	G3	91 (51.4%)	126 (70.4%)	
	G4	0 (0%)	0 (0%)	
Barrett’s esophagus	No	129 (92.8%)	79 (92.9%)	> 0.05
	Yes	10 (7.2%)	6 (7.1%)	

*p < 0.05; **p < 0.01

### Exploratory tumor–normal discrimination and prognostic association of TIMP1 in STAD

**Fig. 4:**
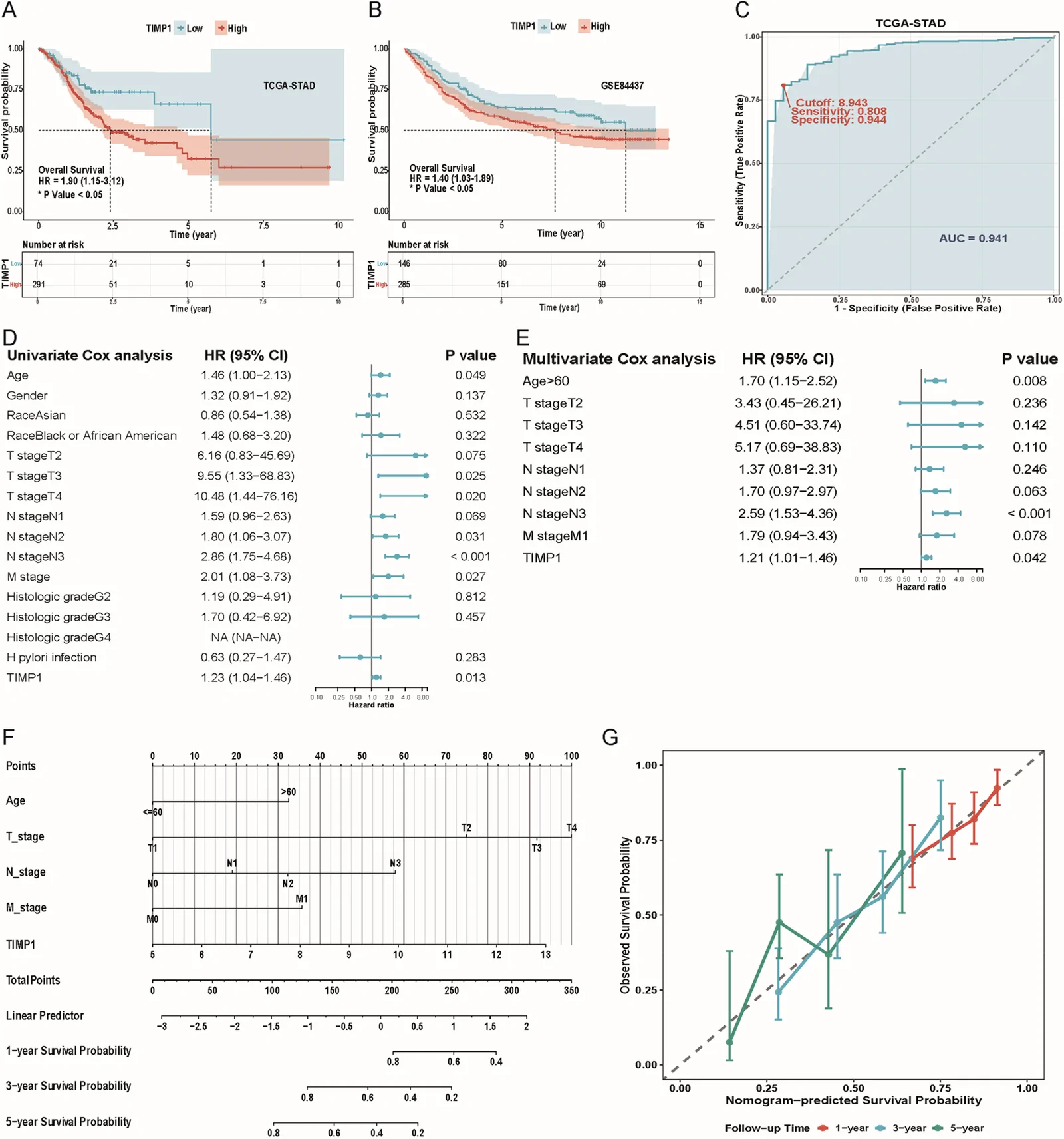
Diagnostic and prognostic performance of TIMP1 in GC. **A**, **B **Kaplan–Meier survival curves for the high and low TIMP1 expression groups in the TCGA_STAD and GSE84437 cohorts. **C **Exploratory ROC analysis of TIMP1 expression in the TCGA_STAD cohort. **D **Forest map based on Univariate Cox analysis for overall survival. **E **Forest map based on Multivariate Cox analysis for overall survival. **F **Nomogram incorporating age, pathological T stage, pathological N stage, pathological M stage and TIMP1 expression to estimate 1-, 3- and 5-year overall survival probabilities. **G **Calibration plots were used to validate the nomogram model. ROC, receiver operating characteristic; AUC, area under the curve; OS, overall survival; HR, hazard ratio; CI, confidence interval; KM, Kaplan–Meier; T stage, tumor stage; N stage, nodal stage; M stage, metastasis stage

Exploratory ROC analysis in the TCGA_STAD cohort showed that TIMP1 expression discriminated GC tissues from normal gastric tissues with an AUC of 0.941 (sensitivity = 0.808; specificity = 0.944; Fig. 4C). Because TCGA_STAD contributed to the initial transcriptomic discovery analysis, this ROC estimate represents within-study apparent discrimination rather than independent diagnostic validation. Kaplan-Meier survival analysis demonstrated that high TIMP1 expression was significantly associated with poorer overall survival (OS) in both the TCGA_STAD cohort (HR = 1.90, *P* < 0.05) and the external validation dataset GSE84437 ( HR = 1.40, *P* < 0.05) (Fig. 4A, B). Univariate and multivariate Cox regression analyses were performed to identify the prognostic determinants of GC in relation to TIMP1 expression. Univariate Cox regression analysis showed that higher TIMP1 expression was associated with poorer OS (HR = 1.23, 95% CI = 1.04–1.46, *P* = 0.013). Age > 60 years was also associated with poorer OS (HR = 1.46, 95% CI = 1.00–2.13, *P* = 0.049). Significant associations were additionally observed for advanced T stage (T3 vs. T1, *P* = 0.025; T4 vs. T1, *P* = 0.020), N stage (N2 vs. N0, *P* = 0.031; N3 vs. N0, *P* < 0.001), and M stage (M1 vs. M0, *P* = 0.027) (Fig. 4D). After multivariable adjustment, higher TIMP1 expression remained significantly associated with poorer OS (HR = 1.21, 95% CI = 1.01–1.46, *P* = 0.042), together with age > 60 years (HR = 1.70, 95% CI = 1.15–2.52, *P* = 0.008) and N3 stage (HR = 2.59, 95% CI = 1.53–4.36, *P* < 0.001) (Fig. 4E).

A nomogram incorporating the variables entered into the multivariable Cox model, including age, pathological T stage, pathological N stage, pathological M stage and TIMP1 expression, was constructed to estimate 1-, 3- and 5-year OS probabilities (Fig. 4F). Calibration curves were used to assess agreement between predicted and observed survival probabilities (Fig. 4G).

### DEG identification and functional enrichment analysis of TIMP1

**Fig. 5:**
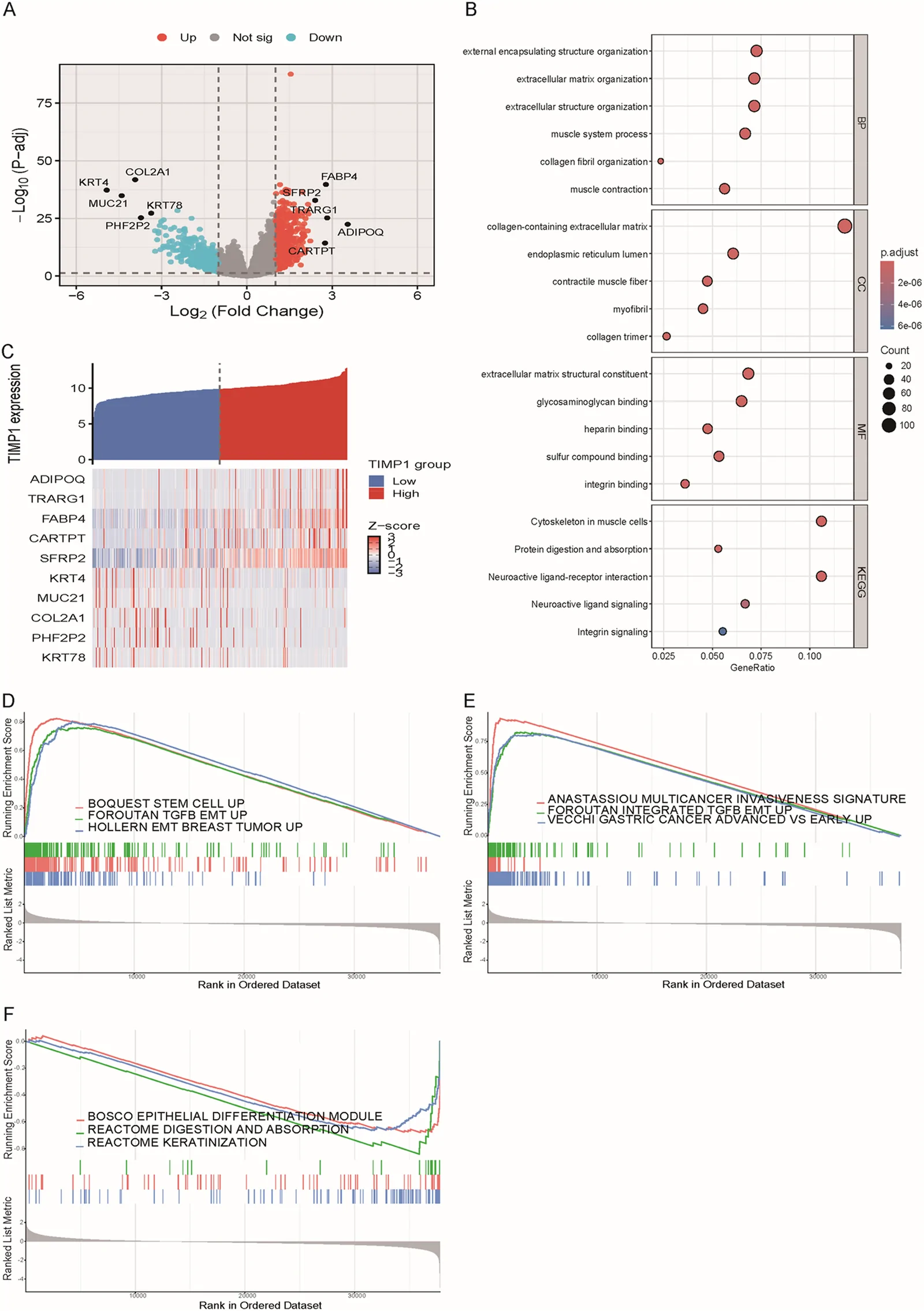
Transcriptomic features and pathway enrichment of TIMP1-high GC. **A **Volcano plot showing the DEGs between the TIMP1-high and TIMP1-low groups. **B **GO and KEGG enrichment analysis of the DEGs, emphasizing ECM organization and integrin signaling. **C **Heat map showing the top five upregulated and downregulated genes with TIMP1 expression. **D**-**F **GSEA results showing enrichment in EMT, TGF-β signaling, and stem cell-related pathways in the high expression group, and epithelial differentiation in the low expression group. GSEA, Gene Set Enrichment Analysis; ECM, Extracellular Matrix; EMT, Epithelial-Mesenchymal Transition. ECM, extracellular matrix; EMT, epithelial–mesenchymal transition; TGF-β, transforming growth factor-β

Between the cohorts with high and low TIMP1 expression, a total of 1,557 transcriptomic genes showed differential expression, including 556 genes that were upregulated and 1,001 genes that were downregulated (with an adjusted p-value < 0.05 and |log2 fold change| >1) (Fig. 5A). The top five upregulated and downregulated differential genes (ADIPOQ, TRARG1, FABP4, CARTPT, SFRP2, KRT4, MUC21, COL2A1, PHF2P2, KRT78) were subjected to further analysis. Heatmaps were employed to delineate the relationship between these genes and TIMP1 expression (Fig. 5C). GO enrichment analysis indicated that the differentially expressed genes (DEGs) were predominantly involved in biological processes (BP), including external encapsulating structure organization and extracellular matrix (ECM) organization. Cellular component (CC) analysis revealed enrichment in the collagen-containing extracellular matrix and endoplasmic reticulum lumen (Fig. 5B). Molecular functions (MF) were associated with integrin binding and ECM structure. Furthermore, KEGG pathway analysis highlighted significant enrichment in protein digestion and absorption pathways (Fig. 5B). GSEA showed that high TIMP1 expression was significantly associated with epithelial–mesenchymal transition (EMT), TGF-β signaling, stem-cell-related pathways and malignant progression-related gene sets (Fig. 5D, E). Conversely, the TIMP1-low group exhibited significant enrichment in the epithelial differentiation module and normal digestion/absorption pathways (Fig. 5F).

### PPI network and functional enrichment analysis of TIMP1-related genes

**Fig. 6:**
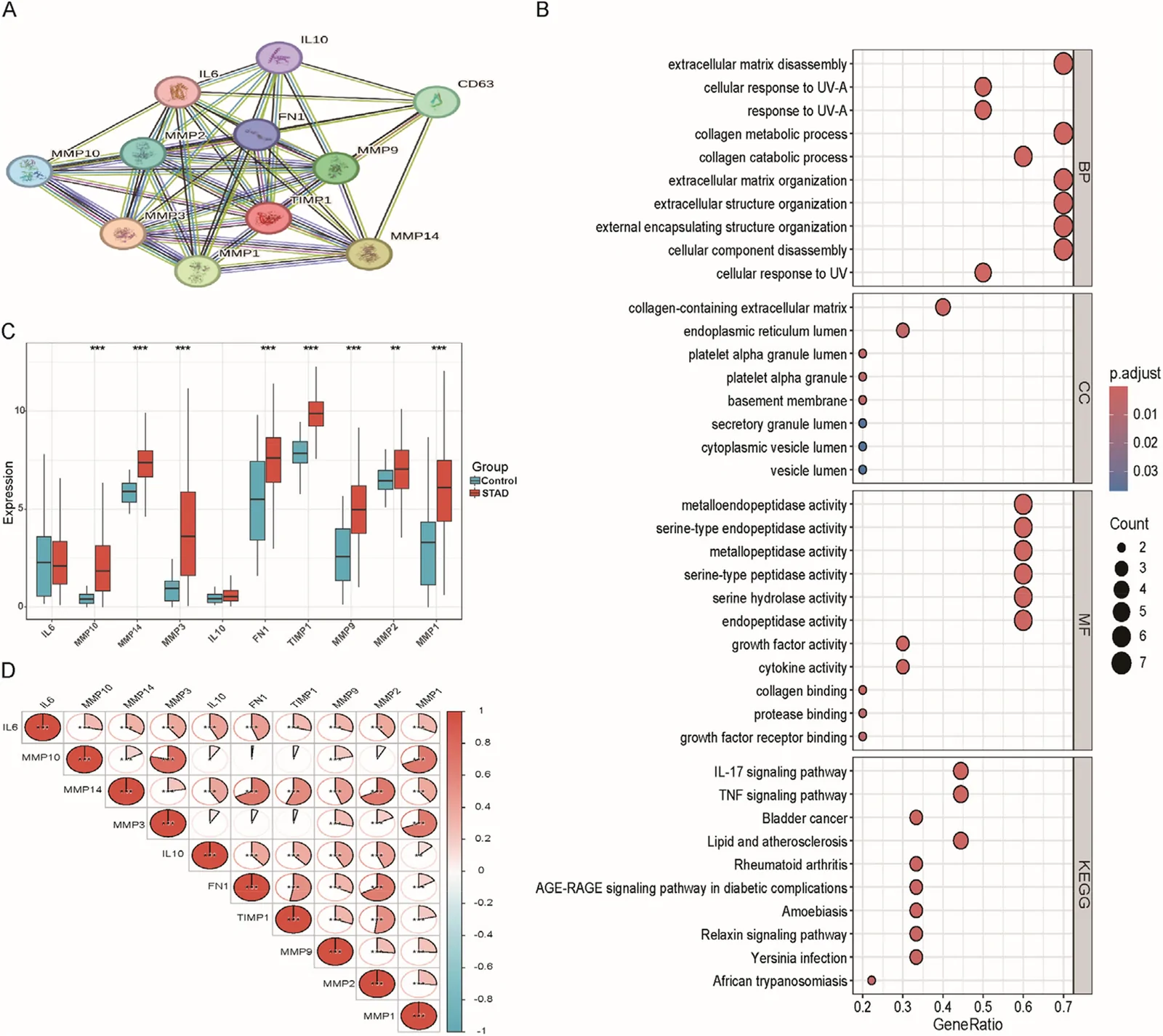
PPI network and functional annotation of TIMP1-associated genes in GC. **A **PPI network of TIMP1-related genes. **B **GO/KEGG functional enrichment analysis of TIMP1-related genes. **C **Expression of TIMP1-related genes in STAD. **D **Correlation between TIMP1 and related genes. *p < 0.05; **p < 0.01; ***p < 0.001; ns, not significant

To explore the molecular partners of TIMP1, a PPI network was constructed using the STRING database. We identified a cluster of genes exhibiting close interactions with TIMP1, including the MMP family (MMP1/2/3/9/10/14), proinflammatory cytokines (IL6 and IL10), and FN1 (Fig. 6A). Validation in the TCGA_STAD dataset confirmed that TIMP1 and nearly all associated candidates were significantly overexpressed in tumor tissues compared to normal controls, with the exception of IL6 and IL10, which showed no significant difference (Fig. 6C). Correlation analysis revealed strong positive associations across the candidate gene sets. Specifically, TIMP1 exhibited the highest correlation with MMP14, followed by MMP2 and FN1 (all *P* < 0.001) (Fig. 6D). Functional enrichment of these TIMP1-related genes focused on ECM disassembly, collagen metabolic processes, and basement membrane organization (Fig. 6B). KEGG analysis indicated that these genes were involved in inflammatory signaling, including the IL-17, TNF, and AGE-RAGE signaling pathways (Fig. 6B). These findings support the notion that TIMP1 is embedded in an ECM-remodeling and inflammation-associated molecular network in GC.

### Immune infiltration analysis

**Fig. 7:**
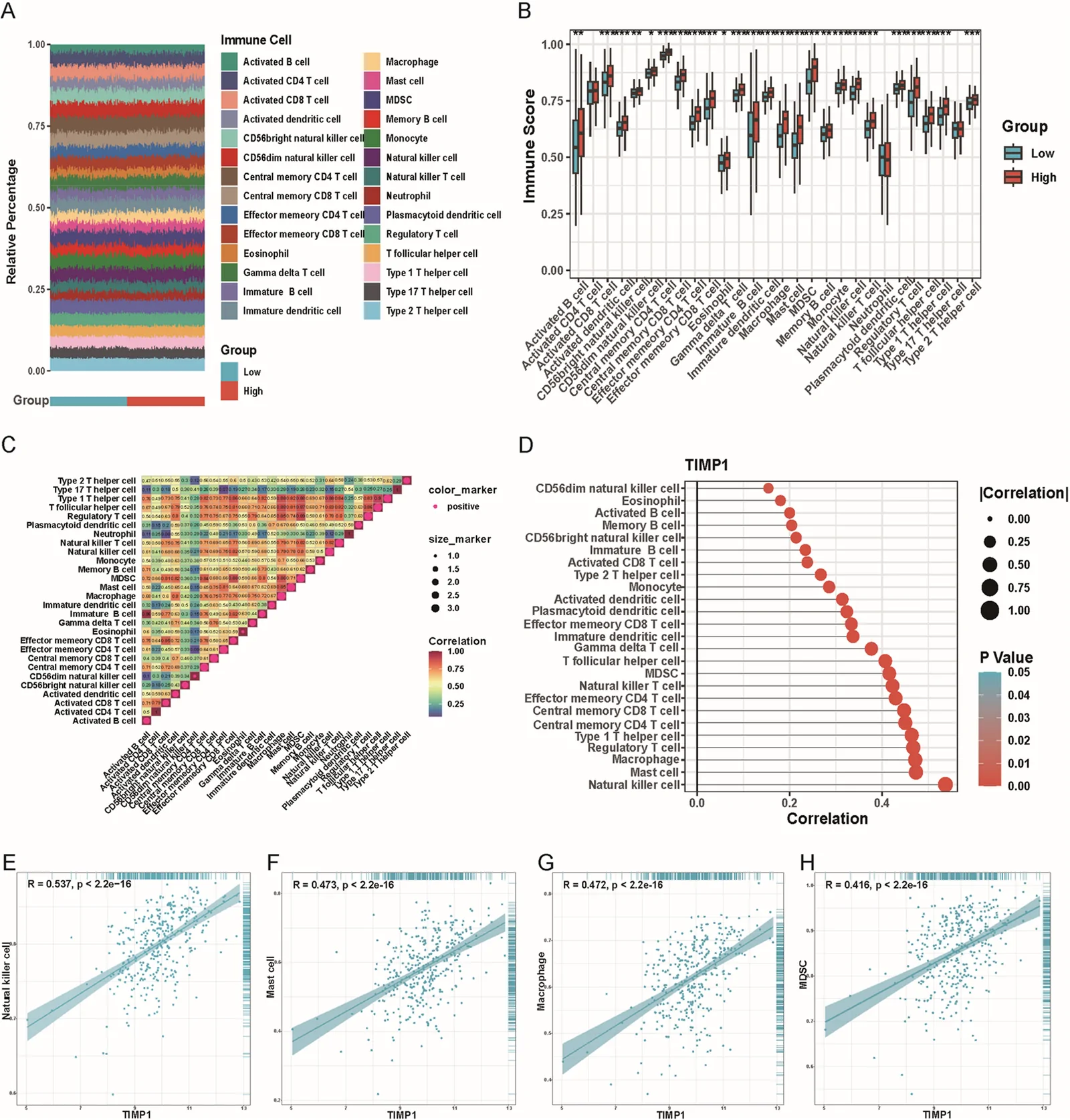
Analysis of immune infiltration. **A **Stacked bar plot of the relative percentage of 28 immune cell types in the high and low TIMP1 groups. **B **Degree of immune infiltration of different immune cells in high and low TIMP1 expression. **C**, **D **Correlation between TIMP1 expression and immune cell infiltration levels. **E**-**H **Scatter plots showing a positive correlation between TIMP1 and specific immune cells, including NK cells, Mast cells, Macrophages, and MDSCs. ssGSEA, single-sample Gene Set Enrichment Analysis; MDSC, Myeloid-derived Suppressor Cells. *p < 0.05; **p < 0.01; ***p < 0.001; ns, not significant

To investigate the relationship between TIMP1 and the tumor immune microenvironment (TIME), we quantified the relative abundance of 28 immune cell types in the TCGA_STAD cohort using the ssGSEA algorithm. Spearman’s correlation analysis showed that TIMP1 expression was significantly and positively correlated with the infiltration levels of all 28 immune cell types (*P* < 0.05) (Fig. 7C, D). Among these, the strongest positive correlations were observed with natural killer (NK) cells (*R* = 0.537, *P* < 2.2e − 16), mast cells (*R* = 0.473, *P* < 2.2e − 16), macrophages (*R* = 0.472, *P* < 2.2e − 16), and myeloid-derived suppressor cells (MDSCs) (*R* = 0.416, *P* < 2.2e − 16) (Fig. 7E–H). Comparison between the TIMP1-high and TIMP1-low groups, stratified by the median, showed that the immune scores of nearly all cell subsets were significantly higher in the high-expression group (*P* < 0.05) (Fig. 7A, B). Although high TIMP1 expression was associated with a highly infiltrated immune phenotype, the concurrent enrichment of immunosuppressive subsets, particularly macrophages and MDSCs, suggests that TIMP1 may be associated with a stromal-rich and immunosuppressive microenvironment in GC.

### Single-cell analysis identifies TIMP1-associated stromal and myeloid remodeling states in GC and AIG

**Fig. 8:**
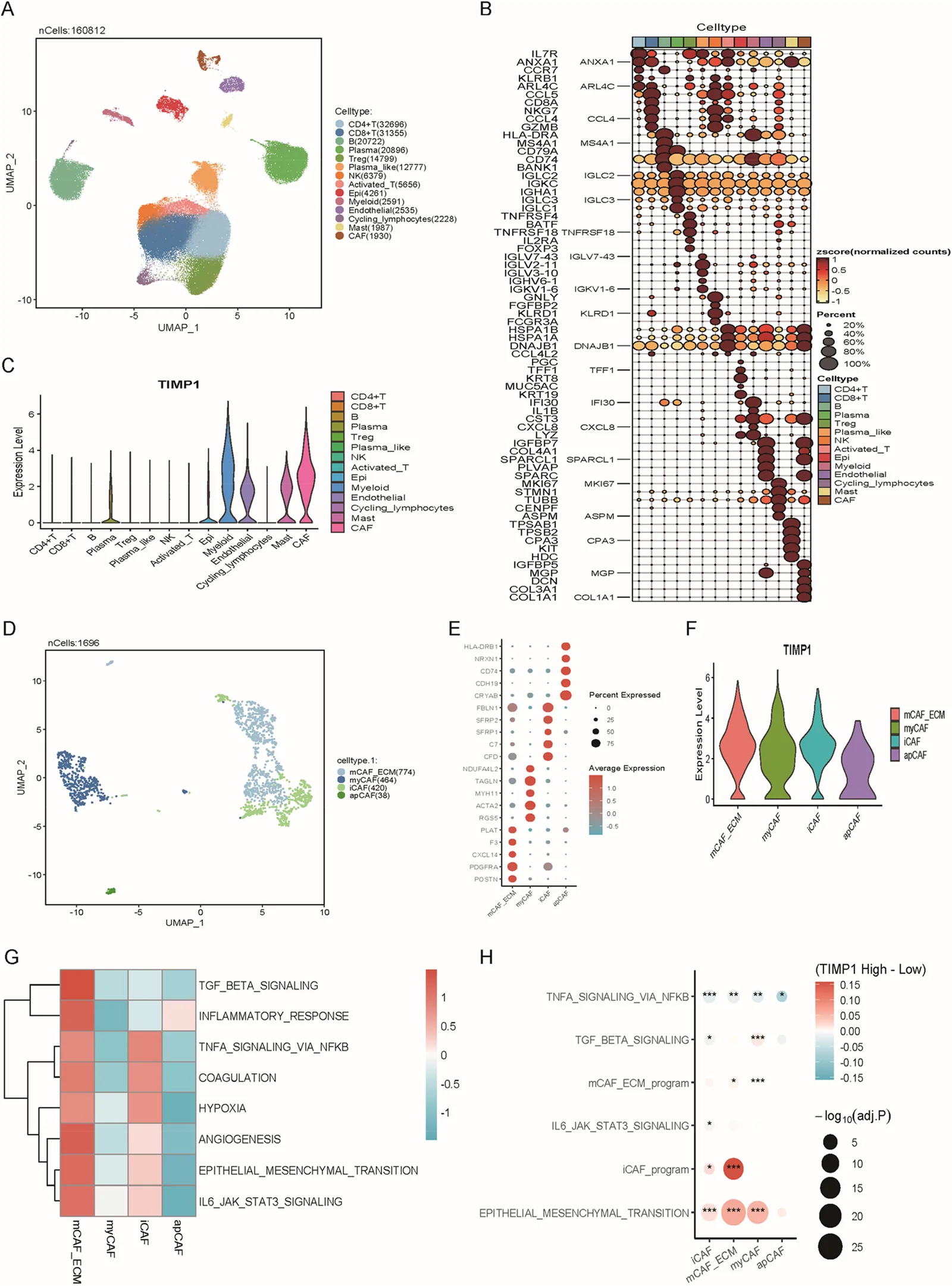
Single-cell mapping of TIMP1-associated CAF states. **A **UMAP visualization of 160,812 single cells from GC tissues colored by major cell type. **B **Dot plot showing representative marker genes for major cell-type annotation. Dot size indicates the proportion of cells expressing each gene, and color indicates scaled average expression. **C **Violin plot showing TIMP1 expression across major cell types. **D **UMAP visualization of reclustered CAFs. **E **Dot plot showing representative marker genes for CAF-state annotation. **F **Violin plot showing TIMP1 expression across CAF states. **G **Heatmap showing selected pathway and functional program activities across CAF states. **H **Bubble plot comparing pathway and program scores between TIMP1-high and TIMP1-low CAFs within each CAF state. Dot color indicates score difference, and dot size indicates -log10 adjusted P value. Asterisks indicate adjusted significance. *p < 0.05; **p < 0.01; ***p < 0.001; ns, not significant. UMAP, uniform manifold approximation and projection; CAF, cancer-associated fibroblast; mCAF_ECM, matrix cancer-associated fibroblast with extracellular matrix program; myCAF, myofibroblastic cancer-associated fibroblast; iCAF, inflammatory cancer-associated fibroblast; apCAF, antigen-presenting cancer-associated fibroblast; TGF-β, transforming growth factor-β; TNFA/NF-κB, tumor necrosis factor-α/nuclear factor-κB; IL6–JAK–STAT3, interleukin-6–Janus kinase–signal transducer and activator of transcription 3; EMT, epithelial–mesenchymal transition

To define the cellular context of TIMP1 expression, we analyzed 160,812 single cells from GC tissues and annotated 14 major cell populations, including epithelial cells, CAFs, myeloid cells, endothelial cells, lymphoid populations, plasma cells and mast cells (Fig. 8A, B). TIMP1 expression was enriched in CAF and myeloid compartments, indicating a predominant microenvironmental expression pattern in GC (Fig. 8C).

We next reclustered 1,696 CAFs and identified four transcriptional states: mCAF_ECM, myCAF, iCAF and apCAF (Fig. 8D, E). TIMP1 was expressed across multiple CAF states, with higher levels in mCAF_ECM, myCAF and iCAF, and relatively lower expression in apCAF (Fig. 8F). Thus, TIMP1 did not define a single CAF subtype, but marked a broader activated CAF compartment. Functional scoring showed that mCAF_ECM displayed higher TGF-β signaling, inflammatory response, TNFA/NF-κB signaling, hypoxia, angiogenesis, EMT and IL6–JAK–STAT3 activity (Fig. 8G). Within-state comparison further showed increased EMT-associated scores in TIMP1-high CAFs across several CAF states, whereas inflammatory and cytokine-related changes were state-dependent rather than uniform (Fig. 8H).

**Fig. 9:**
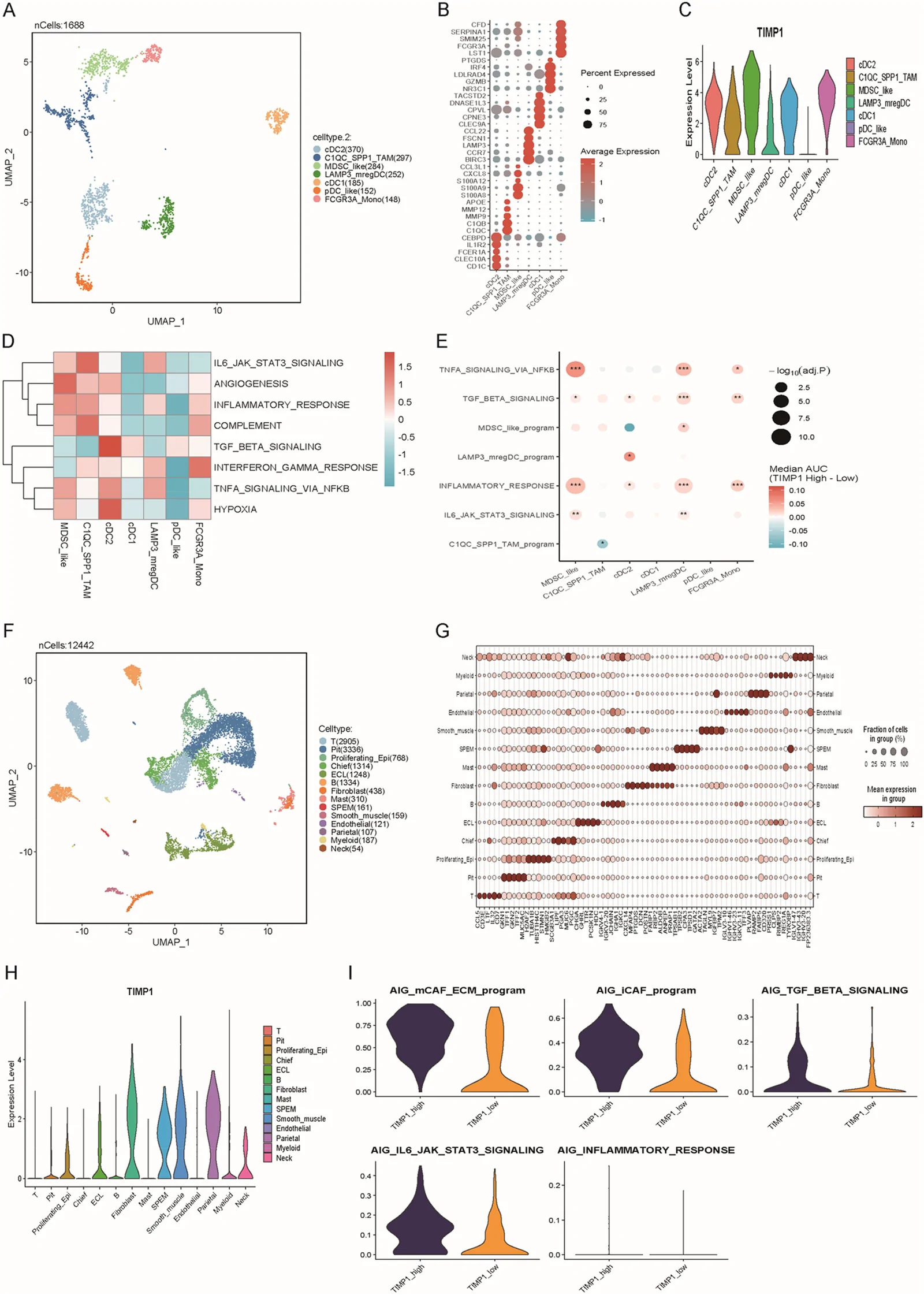
TIMP1-associated myeloid states and fibroblast activation in AIG. **A **UMAP visualization of reclustered GC myeloid cells. **B **Dot plot showing representative marker genes for the myeloid state annotation. Dot size indicates the percentage of cells expressing each gene, and color indicates scaled average expression. **C **Violin plot showing TIMP1 expression across GC myeloid states. **D **Heatmap showing selected pathway and functional program activities across GC myeloid states. **E **Bubble plot comparing pathway and program scores between TIMP1-high and TIMP1-low cells within each myeloid state. Dot color indicates the median AUC difference, and dot size indicates -log10 adjusted P value. Asterisks indicate adjusted significance. **F **UMAP visualization of the AIG scRNA-seq dataset colored by major cell type. **G **Dot plot showing representative marker genes for AIG cell-type annotation. Dot size indicates the fraction of cells expressing each gene, and color indicates mean expression. **H **Violin plot showing TIMP1 expression across major AIG cell types. **I **Violin plots comparing CAF-associated stromal and inflammatory transcriptional program scores between TIMP1-high and TIMP1-low AIG fibroblasts. Asterisks indicate Benjamini–Hochberg-adjusted significance from Wilcoxon rank-sum tests. *p < 0.05; **p < 0.01; ***p < 0.001; ns, not significant. cDC, conventional dendritic cell; pDC, plasmacytoid dendritic cell; TAM, tumor-associated macrophage; MDSC, myeloid-derived suppressor cell; mregDC, mature regulatory dendritic cell; FCGR3A_Mono, FCGR3A-positive monocyte; CAF, cancer-associated fibroblast; mCAF_ECM, matrix cancer-associated fibroblast with extracellular matrix program; iCAF, inflammatory cancer-associated fibroblast; TGF-β, transforming growth factor-β; IL6–JAK–STAT3, interleukin-6–Janus kinase–signal transducer and activator of transcription 3; TNFA/NF-κB, tumor necrosis factor-α/nuclear factor-κB

Because TIMP1 was also enriched in the myeloid compartment, we reclustered 1,688 myeloid cells and identified seven states, including cDC2, C1QC_SPP1_TAM, MDSC_like, LAMP3_mregDC, cDC1, pDC_like and FCGR3A_Mono (Fig. 9A, B). TIMP1 was detected in multiple myeloid states, particularly MDSC_like, C1QC_SPP1_TAM, LAMP3_mregDC and FCGR3A_Mono (Fig. 9C). TIMP1-high MDSC_like cells showed higher TNFA/NF-κB, inflammatory response and IL6–JAK–STAT3 scores, while TIMP1-high LAMP3_mregDC cells showed increased inflammatory, TGF-β, IL6–JAK–STAT3 and MDSC-like programs (Fig. 9D, E). These data link TIMP1-high myeloid cells to inflammatory and immunoregulatory programs, without supporting a single homogeneous myeloid activation state.

We further analyzed the AIG scRNA-seq dataset to examine TIMP1-associated stromal activation in a non-malignant chronic inflammatory gastric setting. A total of 12,442 cells were annotated into epithelial, immune, stromal and endothelial populations (Fig. 9F, G). TIMP1 was detected across several AIG cell types, including stromal-associated compartments (Fig. 9H). Within AIG fibroblasts, TIMP1-high cells showed increased CAF-associated stromal and inflammatory transcriptional program activity. The strongest differences were observed for the mCAF_ECM-like, activated fibroblast-associated and IL6–JAK–STAT3 programs, with additional significant distributional differences in TGF-β signaling and inflammatory-response scores after multiple-testing correction (Fig. 9I). Because AIG is a non-malignant inflammatory condition, these findings were interpreted as fibroblast activation with CAF-associated transcriptional features rather than evidence of bona fide CAFs or a premalignant CAF state.

### inferCNV and CellChat reveal ECM-dominant communication toward inferCNV-defined malignant epithelial populations

**Fig. 10:**
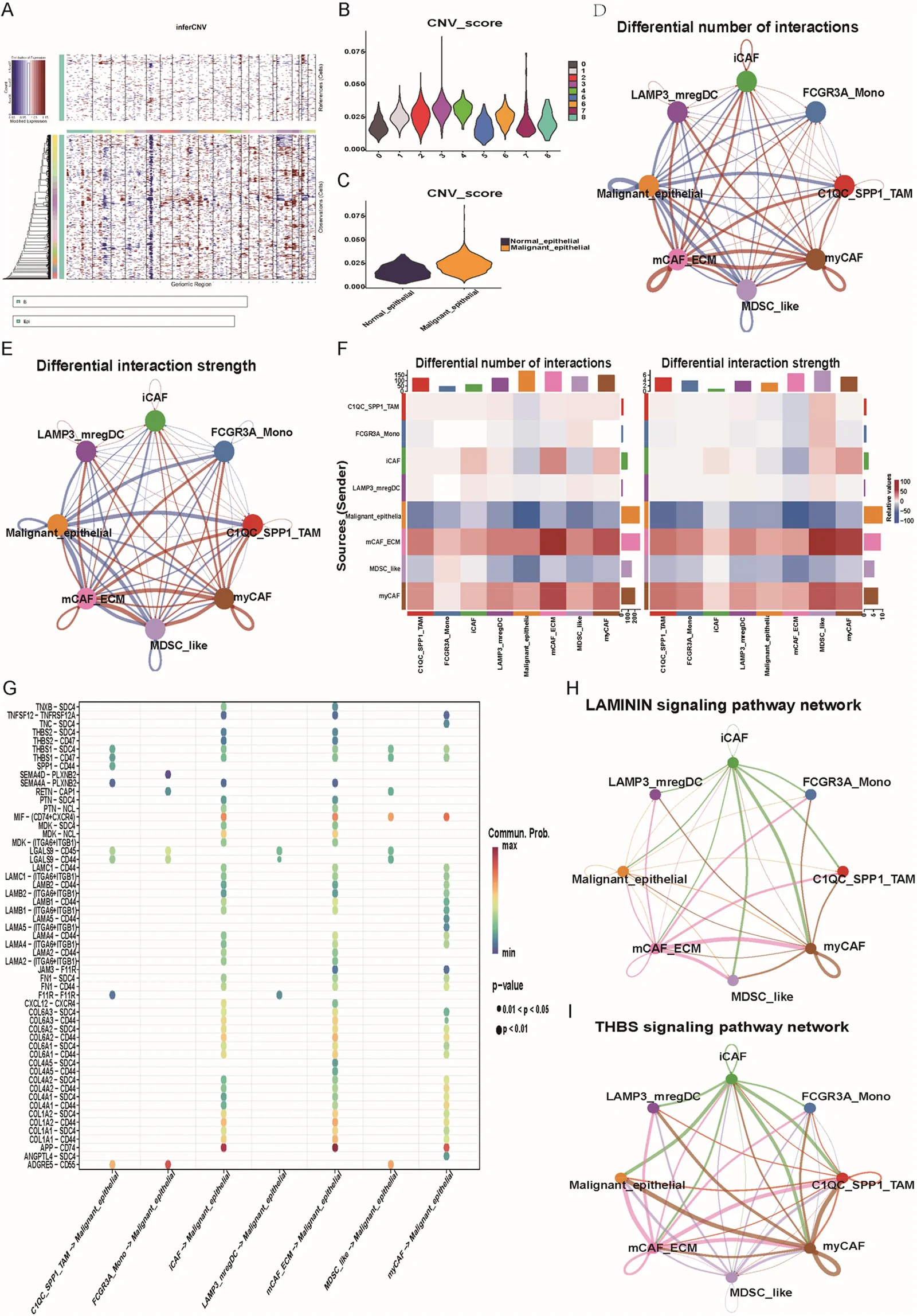
ECM-dominant stromal–myeloid communication. **A **inferCNV heatmap of epithelial cells using B cells as reference cells. Red and blue indicate inferred chromosomal gains and losses, respectively. **B **Distribution of CNV scores across epithelial clusters. **C **Comparison of CNV scores between inferCNV-defined normal epithelial and inferCNV-defined malignant epithelial populations. **D**, **E **Differential CellChat networks showing changes in inferred interaction number (**D**) and interaction strength (**E**). **F **Heatmaps showing differential interaction number and interaction strength across sender–receiver pairs. **G **Bubble plot showing inferred ligand–receptor interactions from CAF and myeloid states to inferCNV-defined malignant epithelial populations. Dot color indicates communication probability, and dot size indicates statistical significance. **H**, **I **Circle plots showing representative LAMININ (**H**) and THBS (**I**) signaling networks involving CAF, myeloid and inferCNV-defined malignant epithelial populations. ECM, extracellular matrix; inferCNV, inference of copy number variation; CNV, copy number variation

To define epithelial receiver populations for downstream communication analysis, we performed inferCNV using B cells as the non-malignant reference population (Fig. 10A). Relative CNV burden varied across epithelial clusters, with cluster 5 exhibiting the lowest relative CNV score (Fig. 10B, C). Following the inferCNV-based operational classification used in previous single-cell GC studies, cluster 5 was classified as the inferCNV-defined normal epithelial population, whereas the remaining epithelial clusters were classified as inferCNV-defined malignant epithelial populations for downstream analyses.

We then used CellChat to examine communication from CAF and myeloid states to inferCNV-defined malignant epithelial receiver populations. Sender populations included mCAF_ECM, iCAF, myCAF, C1QC_SPP1_TAM, MDSC_like, LAMP3_mregDC and FCGR3A_Mono. CellChat network analysis showed extensive stromal–myeloid–epithelial communication, with mCAF_ECM and myCAF contributing prominently to interaction number and strength (Fig. 10D–F).

Ligand–receptor interactions directed toward inferCNV-defined malignant epithelial populations were dominated by ECM and adhesion-related pathways. Representative pairs included COL1A1/2–CD44 or SDC4, COL4A1/2–CD44 or SDC4, COL6A1/2/3–CD44 or SDC4, FN1–CD44 or SDC4, LAMININ ligands with ITGA6–ITGB1 or CD44, SPP1–CD44 and THBS1/2–CD47 or SDC4 (Fig. 10G). LAMININ and THBS signaling also formed broad communication networks involving CAF, myeloid and malignant epithelial populations (Fig. 10H, I). These results indicate that TIMP1-associated stromal and myeloid states are embedded in ECM-dominant communication with inferCNV-defined malignant epithelial populations.

### Spatial transcriptomics maps TIMP1-associated stromal–myeloid remodeling regions in GC

To examine whether TIMP1-associated programs were spatially organized in GC tissues, we projected TIMP1 expression, RCTD-estimated fibroblast and myeloid weights, and scRNA-seq-derived mCAF_ECM, C1QC/SPP1 TAM, MDSC-like and EMT program scores onto two spatial transcriptomic sections.

**Fig. 11:**
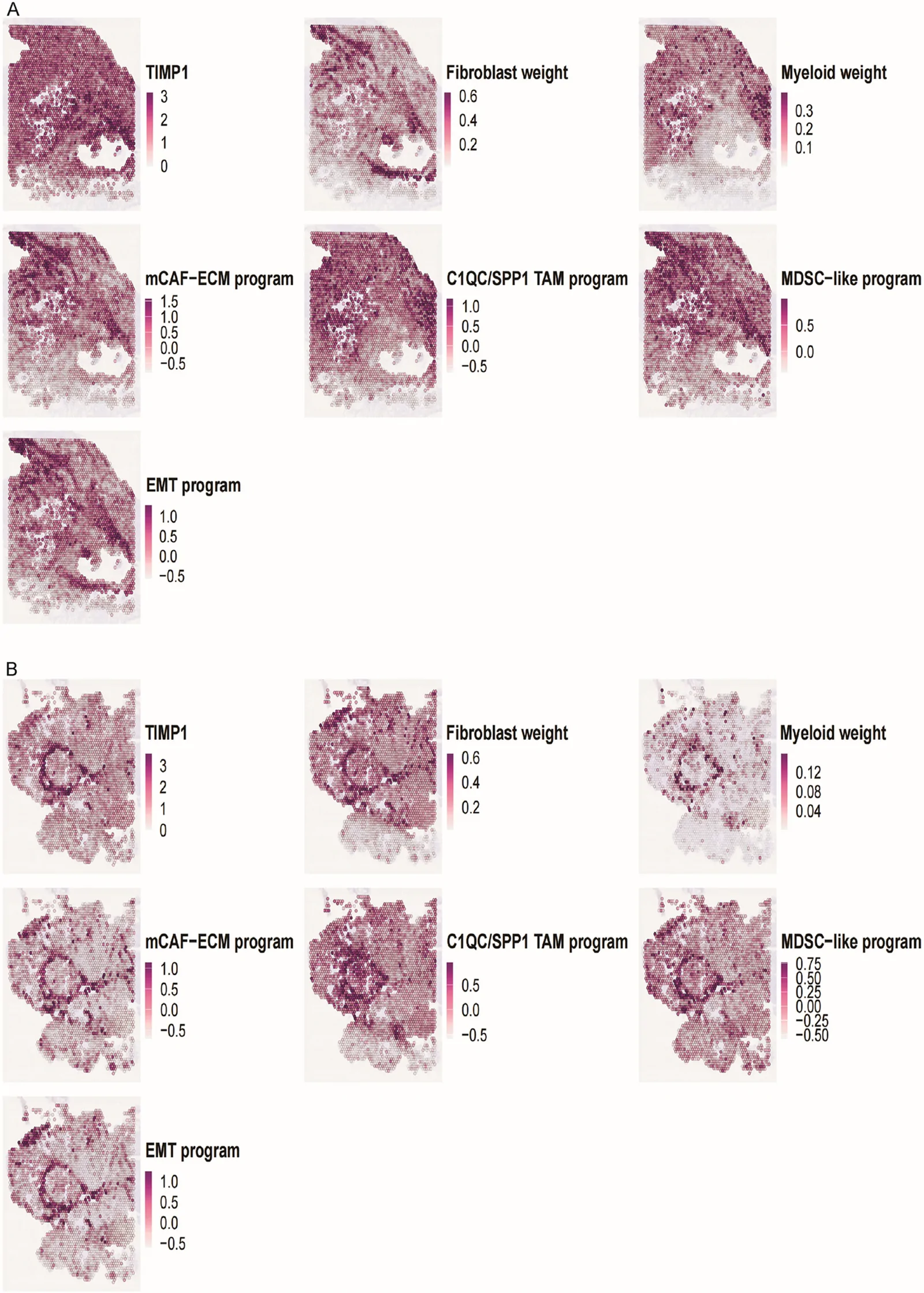
Spatial mapping of TIMP1-associated remodeling niches. **A**, **B **Spatial maps of TIMP1 expression, RCTD-estimated fibroblast and myeloid weights, and scRNA-seq-derived remodeling program scores in two GC spatial transcriptomic samples, s4 (**A**) and s5 (**B**). Projected programs include mCAF_ECM, C1QC/SPP1 TAM, MDSC-like and EMT programs. Spots are overlaid on the corresponding H&E-stained tissue sections. Feature values were capped at the 1st and 99th percentiles for visualization. RCTD, robust cell type decomposition

In sample s4, TIMP1 showed a multifocal and regionally restricted pattern. TIMP1-enriched areas were located near fibroblast-rich stromal regions and partially overlapped with increased myeloid weights and remodeling program scores, including mCAF_ECM, C1QC/SPP1 TAM, MDSC-like and EMT programs (Fig. 11A). Sample s5 showed a related but spatially distinct pattern, with TIMP1 forming a ring-like or band-like distribution aligned with fibroblast-enriched regions. mCAF_ECM and EMT programs were enriched near TIMP1-positive areas, whereas myeloid-related programs were more focal and heterogeneous (Fig. 11B). Together, these spatial patterns support the presence of TIMP1-positive stromal remodeling regions accompanied by myeloid-associated and EMT-related transcriptional programs.

## Discussion

In this study, we used an integrative multi-omics strategy to investigate whether AIG-related inflammatory mucosal remodeling and GC-associated tumor microenvironmental remodeling share molecular and cellular programs. TIMP1 emerged as the only candidate supported by both machine-learning-based feature prioritization and PPI network analysis. Single-cell and spatial analyses further indicated that TIMP1 was mainly associated with CAF and myeloid compartments rather than being restricted to epithelial tumor cells. In AIG, TIMP1-high fibroblasts displayed CAF-associated stromal and inflammatory transcriptional features, whereas in GC, TIMP1-associated stromal and myeloid states were embedded within ECM-dominant remodeling contexts.

The AIG-GC relationship remains biologically plausible but clinically heterogeneous. Prior studies have shown that AIG can induce oxyntic atrophy, hypergastrinemia, epithelial metaplasia and, in selected contexts, gastric neoplasia [7, 10–12]. Recent single-cell work further demonstrated that AIG and H. pylori gastritis can generate comparable cancer-related metaplastic epithelial states, including an ANPEP/CD13-positive incomplete intestinal metaplasia-like population transcriptionally related to GC [13]. However, these studies do not establish that AIG uniformly progresses to adenocarcinoma. Our analysis extends this epithelial-centered view by showing that AIG and GC also share transcriptomic signals related to immune response, gastric functional remodeling and collagen-containing ECM. Thus, AIG-associated inflammatory mucosa may include stromal remodeling features in addition to epithelial metaplastic changes, although longitudinal evidence is still required to determine whether such stromal programs precede cancer development.

The functional profile of the 51 shared DEGs also provides context for interpreting the AIG-GC overlap. Enrichment of the antimicrobial humoral immune response is compatible with chronic mucosal immune activation, whereas enrichment of gastric acid secretion is consistent with the oxyntic mucosal injury that defines AIG [7]. Retinol metabolic processes may be relevant to epithelial differentiation and mucosal remodeling, although this finding should be treated as pathway-level association rather than direct evidence of altered retinoid signaling [35]. The appearance of collagen-containing ECM terms supports a stromal remodeling component and is consistent with emerging evidence that ECM organization shapes GC tissue structure and tumor behavior [36]. By contrast, non-gastric Disease Ontology terms should be interpreted cautiously because they may reflect shared inflammatory, metabolic or tissue-remodeling annotations rather than disease-specific mechanisms.

Importantly, the 51 shared DEGs should not be interpreted as an AIG–GC-specific molecular signature. Without additional inflammatory or non-autoimmune gastritis control cohorts, part of this overlap may reflect broader responses to chronic inflammation, mucosal injury, metabolic change or stromal remodeling. The present analysis therefore supports transcriptomic overlap between AIG and GC rather than disease-specific molecular continuity.

TIMP1 emerged as the central candidate from our discovery framework and showed clinical relevance in GC. Exploratory ROC analysis in TCGA_STAD showed high apparent tumor–normal discrimination (AUC = 0.941); however, because TCGA_STAD contributed to the initial discovery process, this estimate should not be interpreted as independent diagnostic validation. Likewise, the small serum RT-qPCR cohort provides preliminary RNA-level support for elevated circulating TIMP1-associated signal rather than validation of a clinically established serum TIMP1 protein assay. Higher TIMP1 expression was associated with poorer OS in both TCGA_STAD and GSE84437. In the TCGA multivariable Cox analysis, TIMP1 retained significant prognostic association after adjustment for age and the T, N and M components included in the model. Nevertheless, treatment-related variables and detailed histological subtype were not included, and residual clinical confounding cannot be excluded. The machine-learning analyses should likewise be interpreted as feature-prioritization procedures rather than predictive-model validation. Although 10-fold cross-validation was used for LASSO feature selection, no nested cross-validation or independent diagnostic test cohort was used, and the random forest analysis did not incorporate repeated cross-validation or class rebalancing. The resulting feature rankings should therefore be interpreted as exploratory candidate prioritization rather than estimates of clinical predictive performance [37]. Previous work has linked TIMP1 to ECM regulation, matrix metalloproteinase biology, EMT-like phenotypic change, tumor progression, immune modulation and treatment resistance [22, 25–27]. In GC, the SOX9/TIMP1 axis has been reported to affect tumor-cell behavior and dendritic-cell maturation [23, 24]. Compared with these tumor-intrinsic studies, our results suggest that elevated TIMP1 in bulk GC samples may also reflect increased abundance or activation of TIMP1-expressing stromal and myeloid compartments. This interpretation helps reconcile the clinical associations of TIMP1 with its single-cell localization in the tumor microenvironment.

The CAF findings further refine the biological interpretation of TIMP1 in GC. CAFs are now recognized as heterogeneous and plastic stromal cells that can adopt matrix-producing, contractile, inflammatory, antigen-presenting or immunomodulatory programs depending on tissue context and tumor state [16–18]. In our GC scRNA-seq analysis, TIMP1 was not restricted to a single CAF subtype but was expressed across mCAF-ECM, myCAF and iCAF states, with relatively lower expression in apCAF. TIMP1 therefore appears to capture activation across CAF states rather than defining a discrete CAF subtype. Functional scoring showed that TIMP1-positive CAFs were associated with ECM-remodeling and EMT-related programs. This finding is consistent with spatial GC studies showing that fibroblast-enriched regions can coincide with malignant EMT programs and immunosuppressive responses within the tumor microenvironment [6, 21]. Thus, TIMP1-positive CAFs should be interpreted as part of a broad stromal remodeling compartment rather than as a newly defined CAF lineage.

The myeloid compartment provided an additional layer of evidence linking TIMP1 to immunoregulatory remodeling. TIMP1 was detected across several transcriptionally distinct myeloid states, including MDSC_like, C1QC_SPP1_TAM, LAMP3_mregDC and FCGR3A_Mono. TIMP1-high cells showed enrichment of inflammatory and immunoregulatory programs involving TNFA/NF-κB, IL6–JAK–STAT3 and TGF-β-related signaling. These pathways are increasingly recognized as components of the signaling networks that shape antitumor immunity and therapeutic response [38]. However, the MDSC-like and regulatory dendritic-cell annotations in the present study are transcriptional and do not directly establish suppressive function. In our bulk immune infiltration analysis, TIMP1 expression correlated positively with multiple immune cell types, especially macrophages and myeloid-derived suppressor cell-like populations. These findings indicate that TIMP1-high GC should not be interpreted simply as an antitumor immune-hot phenotype. A more balanced interpretation is that TIMP1-high tumors may represent an immune-infiltrated but immunoregulatory microenvironment enriched for remodeling-associated myeloid programs [4, 20].

A notable observation was the presence of TIMP1-high fibroblasts with enhanced stromal and inflammatory transcriptional programs in AIG mucosa. These fibroblasts showed higher activity of CAF-associated ECM-remodeling and activated fibroblast programs together with IL6–JAK–STAT3-related activity. Because AIG is a non-malignant chronic inflammatory condition, these cells should not be classified as bona fide CAFs, nor do the present cross-sectional data establish a premalignant transition toward a CAF state. Instead, they are more conservatively interpreted as activated fibroblasts exhibiting CAF-associated transcriptional features in chronically inflamed gastric mucosa. The AIG single-cell dataset contained only three individuals; therefore, the present cell-level analysis cannot establish patient-level reproducibility or determine whether this fibroblast state is associated with future cancer risk. Validation in larger independent AIG cohorts with longitudinal clinical information will be required. This conservative interpretation also aligns with emerging views that AIG pathogenesis involves cellular crosstalk among epithelial, immune, endocrine and stromal compartments rather than parietal-cell loss alone [39].

CellChat and spatial transcriptomic analyses placed TIMP1-associated states within an ECM-dominant niche. CellChat analysis placed TIMP1-associated stromal and myeloid states within an ECM- and adhesion-dominant interaction context, including COLLAGEN-CD44/SDC4, FN1-CD44/SDC4, LAMININ-ITGA6-ITGB1/CD44, SPP1-CD44 and THBS-CD47/SDC4. These pathways are biologically plausible because ECM-receptor and adhesion-related interactions can influence epithelial plasticity, invasion and immune-cell localization in tumors [16, 19, 36]. Importantly, TIMP1 itself was not identified as the direct ligand-receptor mediator in the CellChat network. Therefore, TIMP1 is best interpreted as a marker of stromal and myeloid states embedded in ECM-dominant communication, rather than as the inferred signaling ligand driving these interactions. Spatial transcriptomics provided regional support for this interpretation by showing that TIMP1-enriched areas were located near fibroblast-rich stromal regions and accompanied by myeloid-associated, mCAF_ECM, MDSC-like and EMT-related programs. However, because Visium-based spatial transcriptomics has spot-level resolution, these data support regional co-localization rather than direct cell-cell contact [40].

The niche-level interpretation of our study is particularly consistent with recent spatial transcriptomic work in GC. Lee et al. used Visium spatial transcriptomics to define spatial compartments within GC tissues and showed that fibroblast-enriched tumor microenvironments upregulate EMT programs in malignant cells and immunosuppressive responses in tumor microenvironment cells [6]. The same study further demonstrated that CCL2-positive fibroblasts and STAT3-activated macrophages are spatially co-localized, associated with unfavorable prognosis and experimentally linked to myeloid recruitment and macrophage STAT3 activation [6]. Our data suggest that TIMP1-associated CAF and myeloid states represent another ECM-remodeling and immunoregulatory stromal context within GC tissues. Thus, TIMP1 may help mark fibroblast–myeloid remodeling regions rather than functioning as a standalone driver of these niches.

Two apparent discrepancies deserve comment. First, protein-level validation showed TIMP1 staining in tumor tissues, whereas scRNA-seq localized TIMP1 mainly to CAF and myeloid compartments. This is not necessarily contradictory. Bulk RNA-seq and immunohistochemistry measure composite tissue signals and may capture both malignant and non-malignant compartments, while scRNA-seq is more sensitive to cellular source but may be affected by sampling, dissociation and dropout. Spatial transcriptomics helps bridge these layers by showing that TIMP1-enriched regions were mainly organized around fibroblast-rich remodeling areas. Second, TIMP1 showed exploratory tumor–normal discrimination, but this should not be interpreted as evidence of a standalone diagnostic classifier. Its association with survival was reproducible across cohorts; nevertheless, GC prognosis remains strongly shaped by clinical stage, nodal status, treatment and molecular heterogeneity [1–5]. Thus, TIMP1 may be most useful as part of a broader clinical and microenvironmental stratification framework.

Several aspects strengthen the present analysis. First, TIMP1 was not selected from a single dataset or algorithm but emerged through convergence across shared transcriptomic alterations, machine-learning-based feature prioritization and PPI network analysis. Second, its clinical association was examined across public transcriptomic cohorts, serum RT-qPCR, HPA-based protein evidence and survival analyses. Third, single-cell analysis resolved the major cellular sources of TIMP1, whereas CellChat and spatial transcriptomics placed TIMP1-associated states within communication and tissue-architecture contexts. Such cross-scale integration can help distinguish reproducible molecular signals from cohort- or platform-specific observations and improve biological interpretation of candidate determinants across complex tumor systems [41]. This multi-layered strategy is consistent with recent GC studies that combine single-cell profiling, spatial mapping, bulk validation and computational feature selection to move from marker discovery to biological interpretation [5, 33].

Several limitations should also be acknowledged. First, most transcriptomic analyses were based on retrospective public cohorts. In particular, the AIG bulk-transcriptomic discovery cohort contained only 14 AIG samples and 9 normal controls, which limits statistical power and generalizability despite the use of multiple-testing-adjusted significance thresholds. Bulk RNA-seq and microarray data are influenced by tumor purity, cell-type composition, intratumoral heterogeneity, platform-related variation and incomplete clinical annotation [42]. These factors may contribute to between-cohort variation and limit causal or cell-specific interpretation of bulk transcriptomic associations. Second, the AIG scRNA-seq dataset contained only three individuals, and the TIMP1-associated fibroblast activation pattern requires validation in larger AIG cohorts with detailed H. pylori history, intestinal metaplasia subtype, gastrin levels and long-term clinical follow-up. Third, CellChat provides computational ligand-receptor inference from transcriptomic data and cannot prove functional signaling [34]. Fourth, Visium-based spatial transcriptomics has spot-level resolution and cannot establish direct cell-cell contact or protein-level interaction [40]. Spatial deconvolution also relied on an scRNA-seq reference from an independent cohort, which may introduce cross-cohort and platform-related uncertainty. Fifth, MDSC-like and CAF-associated transcriptional programs were identified computationally, but their suppressive or remodeling functions were not experimentally validated. In addition, inferCNV provides an expression-based estimate of large-scale chromosomal alterations rather than direct DNA-level confirmation of malignancy. The epithelial labels used in this study should therefore be interpreted as operational inferCNV-based classifications, and low-CNV malignant clones cannot be completely excluded. Future studies incorporating orthogonal genomic profiling would allow more definitive malignant-cell classification. Finally, functional perturbation of TIMP1 was not performed. Accordingly, the associations between TIMP1 expression, fibroblast activation, myeloid remodeling, ECM-related programs and epithelial communication should not be interpreted as evidence of direct regulation. Future studies using TIMP1 knockdown or overexpression, stromal–myeloid co-culture systems, multiplex imaging and spatial protein-level approaches will be required to test these relationships mechanistically.

Despite these limitations, this study provides a microenvironment-centered framework for understanding TIMP1 across AIG and GC. Clinically, TIMP1 may help identify GC patients with stromal-rich, myeloid-enriched and ECM-remodeling tumors. In AIG, TIMP1-associated fibroblast activation may represent a candidate stromal feature for future longitudinal investigation, although its relationship with subsequent neoplastic risk remains unknown. Future clinical studies should establish longitudinal AIG cohorts with standardized documentation of H. pylori exposure, intestinal metaplasia subtype, gastrin levels and neoplastic outcomes. Overall, our findings support TIMP1 as a stromal–myeloid remodeling-associated candidate marker across AIG and GC and propose a testable framework in which AIG-associated fibroblast activation and GC-associated CAF–myeloid niches may represent related, but not necessarily sequential, microenvironmental states.

## Conclusions

In summary, our study supports TIMP1 as a candidate marker associated with stromal–myeloid remodeling across AIG-related mucosal remodeling and GC. TIMP1 was upregulated in GC tissues and serum and showed exploratory diagnostic and prognostic associations. Single-cell analyses localized TIMP1 predominantly to CAF and myeloid compartments, while TIMP1-high AIG fibroblasts displayed CAF-associated stromal and inflammatory transcriptional features. Together, these findings support a hypothesis-generating framework in which TIMP1-associated stromal and myeloid programs may reflect related microenvironmental remodeling states across chronic inflammatory gastric mucosa and GC. Prospective clinical cohorts and functional studies are required to clarify the mechanistic and clinical significance of these associations.

## Supplementary Information

Supplementary Material 1.

## Data Availability

No datasets were generated or analysed during the current study.

## Abbreviations

AIG: Autoimmune gastritis
ANPEP/CD13: Alanyl aminopeptidase / cluster of differentiation 13
apCAF: Antigen-presenting cancer-associated fibroblast
AUC: Area under the curve
BP: Biological process
CAF: Cancer-associated fibroblast
CAFs: Cancer-associated fibroblasts
CC: Cellular component
cDC1: Conventional dendritic cell 1
cDC2: Conventional dendritic cell 2
CNV: Copy number variation
DEG: Differentially expressed gene
DEGs: Differentially expressed genes
DO: Disease Ontology
ECM: Extracellular matrix
EMT: Epithelial–mesenchymal transition
FAK: Focal adhesion kinase
FAP: Fibroblast activation protein
FCGR3A_Mono: FCGR3A-positive monocyte state
FDR: False discovery rate
FN1: Fibronectin 1
GAPDH: Glyceraldehyde-3-phosphate dehydrogenase
GC: Gastric cancer
GEO: Gene Expression Omnibus
GO: Gene Ontology
GSEA: Gene Set Enrichment Analysis
H&E: Hematoxylin and eosin
HPA: Human Protein Atlas
HR: Hazard ratio
IHC: Immunohistochemistry
iCAF: Inflammatory cancer-associated fibroblast
IL6: Interleukin 6
IL10: Interleukin 10
JAK: Janus kinase
KEGG: Kyoto Encyclopedia of Genes and Genomes
LAMP3_mregDC: LAMP3-positive mature regulatory dendritic cell state
LASSO: Least absolute shrinkage and selection operator
MCC: Maximal clique centrality
MDSC: Myeloid-derived suppressor cell
MDSC_like: Myeloid-derived suppressor cell-like state
MF: Molecular function
MMP: Matrix metalloproteinase
mCAF_ECM: Extracellular-matrix-associated matrix cancer-associated fibroblast state
myCAF: Myofibroblastic cancer-associated fibroblast
NF-κB: Nuclear factor kappa B
NK: Natural killer
OS: Overall survival
PCA: Principal component analysis
PCs: Principal components
pDC: Plasmacytoid dendritic cell
pDC_like: Plasmacytoid dendritic cell-like state
PPI: Protein–protein interaction
qPCR: Quantitative polymerase chain reaction
RCTD: Robust cell type decomposition
RF: Random forest
RNA: Ribonucleic acid
ROC: Receiver operating characteristic
RT-qPCR: Reverse transcription quantitative polymerase chain reaction
scRNA-seq: Single-cell RNA sequencing
SDC4: Syndecan 4
SPP1: Secreted phosphoprotein 1
ssGSEA: Single-sample Gene Set Enrichment Analysis
STAD: Stomach adenocarcinoma
STAT3: Signal transducer and activator of transcription 3
TCGA: The Cancer Genome Atlas
TGF-β: Transforming growth factor beta
THBS: Thrombospondin
TIME: Tumor immune microenvironment
TIMP1: Tissue inhibitor of metalloproteinases 1
TME: Tumor microenvironment
TNF: Tumor necrosis factor
TNFA: Tumor necrosis factor alpha
TPM: Transcripts per million
UMAP: Uniform manifold approximation and projection
UMI: Unique molecular identifier
