# Supplementary material for: Multi-omics mapping of TIMP1-associated stromal–myeloid remodeling in autoimmune gastritis and gastric cancer
Source: Hereditas. 2026 Sep 10;163:99. doi: 10.1186/s41065-026-00736-0 (PMC13617772; doi:10.1186/s41065-026-00736-0)
Supplement: Supplementary file 1 [file 41065_2026_736_MOESM1_ESM.docx]

Supplementary Material 1:

**Figure S1 Machine-learning-based feature prioritization in GSE29272 and GSE54129.** (A, B) LASSO regression analysis for feature selection in the GSE29272 dataset. (C, D) LASSO regression analysis in the GSE54129 dataset. (E, F) Ranking of gene importance using the Random Forest (RF) algorithm in GSE29272 and GSE54129, respectively.
